# Bioinformatics based design of thermostable virus-like particles-based vaccine for foot-and-mouth disease serotype A and *in-vivo* evaluation in guinea pigs

**DOI:** 10.3389/fcimb.2026.1760751

**Published:** 2026-03-30

**Authors:** Deepak Praveen Raj Selvaraj, Paramasivam Saravanan, Aparna Madhavan, Das Adwitiya, Chandan Tilak, Beeragere Parameshwaraiah Sreenivasa, Shreya Gopinath, Sekar Elackiya, Saravanamuthu Thiyagarajan, Pallab Chaudhuri

**Affiliations:** 1 Foot-and-Mouth Disease Virus Production (FMDVP) Lab, Indian Council of Agricultural Research (ICAR)-Indian Veterinary Research Institute (IVRI), Bengaluru, Karnataka, India; 2 Division of Structural Bioinformatics, Institute of Bioinformatics and Applied Biotechnology (IBAB), Bengaluru, Karnataka, India

**Keywords:** Virus-like particles, Immunogenicity, *In -silico* bioinformatics, FMDV serotype A/IND/40/2000, Guinea pig challenge studies, Vaccine

## Abstract

**Background:**

Foot-and-mouth disease (FMD) is a contagious disease affecting cloven footed animals. Existing vaccines although effective, has limitations of short immunity, needs cold chain maintenance, high biosafety levels facilities for production and inability to differentiate infected from vaccinated animals. Virus-like particles (VLPs) provide an alternative strategy and the structural integrity of VLPs at moderate temperatures or acidic pH plays major role as they destabilize the capsid and exhibits poor immunogenicity when used as vaccine. Stabilized capsids are developed using bioinformatics approach to predict amino acids in the structural proteins of the FMD virus, which may provide thermostability and can withstand live virus challenge.

**Methods:**

Baculovirus expression system offer an attractive method for producing VLPs of foot-and-mouth disease virus, which mimics the native virus and elicits protective immune response. *In-silico* bioinformatics structural analysis was harnessed to predict thermostable amino acids on the VP2 and VP3 proteins of FMDV serotype A/IND/40/2000 by employing molecular modelling. Consequent mutations were introduced into the VP2 and VP3 proteins, F62Y and H142D, respectively, resulting in the formation of a double mutant (AM-3) and VLPs expressed in Tn5 cells. The AM-3 VLPs were tested in thermostability test *in-vitro* for different temperature and time points and also tested by *in-vivo* in guinea pigs for vaccine efficacy.

**Results:**

The demonstration of capsids of FMDV in the transmission electron micrograph confirmed the expression of AM-3 VLPs. Thermostability studies revealed AM-3 had significantly low degradation (62.5%) on 15 days post storage at 37°C amongst all VLPs and validated by *in-vivo* studies. Finally, the VLPs conferred 90% protection in guinea pigs and could serve as a thermostable candidate vaccine.

**Conclusion:**

The thermostable AM-3 VLPs produced based on bioinformatics without compromising the structural integrity, could confer protective immunity in preclinical studies and which can serve as a potential thermostable candidate vaccine for FMDV serotype A/IND/40/2000 for controlling FMD in ruminants.

## 1 Introduction

Foot-and-mouth disease virus (FMDV), the causative agent of FMD, belonging to the genus *Aphthovirus* of the *Picornaviridae* family, is a highly contagious disease of the cloven-hoofed animals. It causes huge economic loss in ruminants in terms of productivity and reproductive losses which needs to be tackled by control program. The diversity of FMDV exists in the form of seven serotypes (O, A, C, Asia1, SAT1, SAT2, and SAT3) and numerous subtypes (Bachrach, 1968). The FMD vaccine contains binary ethylene imine (BEI) inactivated virus (Bahnemann, 1990) of prevailing serotypes (O, A and Asia1 in India) with oil adjuvants and being used in the control program. The limitations of the inactivated trivalent FMD vaccine include short duration of immunity, stringent bio-containment facility for vaccine manufacturing, maintenance of cold chain, and vaccine related outbreaks. Further, the thermolabile nature of the whole virion disintegrates, leading to poor immunogenicity, warranting deployment of new strategies to improve vaccine stability by maintaining the structural integrity of 146S antigen (virus capsid with inactivated RNA genome is referred as 146S antigen, where S indicates Svedberg unit) (Scott et al., 2017) of the FMDV through bioinformatics approach.

The genome of FMDV consists of P1, P2 and P3 polyproteins. P1 encompasses structural proteins such as VP1, VP2, VP3 and VP4; whereas P2 and P3 encode the non-structural proteins which are responsible for virus replication and translation. P1 and 2A (cleavage protein) together form the empty capsid. The capsid of FMDV is composed of 60 copies each of the structural proteins VP1, VP2, VP3, and VP4. The four structural proteins (VP1-4) together form a single protomer (5S), five protomers join to form a pentamer (12S), and 12 such pentamers combine to form an empty capsid or virus-like particles (VLPs) of 75S which elicits antigenicity and immunogenicity as that of whole virus (Rincón et al., 2014; Cao et al., 2009). The interpentameric (IP) region consists of eight chains of peptides, two each of VP1, VP2, VP3, and VP4. Improving the stable interactions between the peptide chains at the IP interface could offer stability to capsid and prevent its dissociation into intermediates (Kotecha et al., 2015). Earlier VLPs with improved stability were produced by mutating residues at the IP region (Porta et al., 2013). Mutagenic studies revealed that H142 of VP3 is essential for genome uncoating and remains conserved among the serotypes of FMDV (Curry et al., 1995) as it is the major site of electrostatic repulsion for dissociation of capsid under low pH (van Vlijmen et al., 1998). A single mutation of A65H in VP2 in the infectious FMDV serotype C-S8c1 strain increased the thermostability by optimizing the electrostatic interactions (Rincón et al., 2014).

The development of thermostable VLPs is a potential method for optimizing the stability of the vaccine than the infectious virus, as suggested earlier by the introduction of H93C of VP2 mutation at the IP region of FMDV serotype A22 Iraq (Porta et al., 2013). Earlier attempts to predict the mutations by bioinformatics studies for FMDV VLPs of Asia1/IND/63/1972 successfully showed increase thermal stability amongst the mutated VLPs (Aparna et al., 2024). Although, A22 Iraq strain shares high homology with A/IND/40/2000 (88%), interpentameric region of both the strains are less similar to each other which need to be confirmed with thermostability studies. Therefore, we envisaged developing thermostable mutant VLPs of FMDV serotype A/IND/40/2000 using bioinformatics predictions to validate the stability of VLPs through *in-vitro* and *in-vivo* methods. The mutated stable VLPs produced in insect cells were confirmed in western blot and transmission electron microscopy imaging. The thermostability of VLPs was assessed based on shelf-life studies and differential scanning fluorescence assay. We have successfully demonstrated for the first time the protective antibody titer of the wild type and thermostable VLPs of FMDV serotype A in guinea pigs. The challenge studies further confirmed the protective efficacy of the thermostable VLPs as candidate vaccine and also favors the use of differentiation of infected from vaccinated animals (DIVA) as guinea pigs immunized with VLPs did not show 3AB antibodies in the serum samples by blocking ELISA.

Our results suggested that a double mutant, F62Y:H142D (F62Y mutation in VP2 and H142D in VP3) designated as AM-3 VLPs produced in insect cells had shown better thermostability and immunogenicity than the wild type and other mutant VLPs. The bioinformatics-based design of stable capsid has a potential for developing a safe and alternative FMD vaccine, which will be highly preferred in the final stages of the progressive control pathway (PCP) of FMD as per the World Organization for Animal Health (WOAH) guidelines. For prevention and eventual disease eradication, thermostable VLPs based screening ELISAs and vaccines will be highly preferable over the inactivated virus due to their inherent non-infectious nature. Here, we report the innovative bioinformatics approach used in predicting the mutations and eventually validating the *in-silico* methods by including appropriate positive and negative controls. The results of bioinformatic analysis were validated by producing VLPs in insect cells and tested both *in-vitro* and *in-vivo* and could serve as an alternative to current inactivated FMD vaccine for resource limited region for FMD control and eradication.

## 2 Material and methods

### 2.1 Viruses and cells

FMDV serotype A/IND/40/2000 virus (P_10_) and Sf-21 (*Spodoptera frugiperda*) and Tn5 (*Trichoplusia ni*) insect cells available at ICAR - IVRI, Bengaluru, were used to produce VLPs. The Baby Hamster Kidney-21 cell line (BHK-21) procured from the American Type Culture Collection (ATCC, USA) was cultured in Glasgow’s modified Eagle’s medium (HiMedia, India) supplemented with 10% fetal bovine serum (HyClone™, USA) was used for virus production.

### 2.2 Construction of transfer plasmids

The P1-2A-3C region of FMDV serotype A/IND/40/2000 was cloned in pFastbac1 vector as reported earlier (Bhat et al., 2013; Hassine et al., 2020; Gashti et al., 2024). Briefly, RNA was isolated from virus, cDNA was synthesized, P1-2A and 3C genes were amplified separately (**Supplementary Table 1**). The amplified genes were subsequently cloned into the pFastbac1 vector (Invitrogen, USA). The 3C gene of FMDV was mutated at positions 38 and 48 (G38SF48S) by site-directed mutagenesis, and a single cassette of P1-2A-3C was further cloned and inserted into a transfer pFastbac1™ plasmid (pFastbac1-A-P1-2A-3C).

### 2.3 *In-silico* prediction modeling using bioinfomatics to determine the amino acids contributing to the stability of capsids

Homology modelling (HM) of the IP region of the FMDV serotype A/IND/40/2000 (Accession no. HM854025) was constructed using A22 Iraq crystal structure (PDB code: 4GH4) as the template using PRIME module of the Schrodinger software suite interfaced with Maestro module (Schrödinger, 2019). Intra and inter-molecular interactions at the IP region were visualized and analyzed using the PyMol (www.pymol.org) software. *In-silico* point mutations were introduced using ‘coot’ and the rotamer orientation with minimal clashes were used for structure stability predictions.

Molecular dynamic (MD) simulations were performed using the Maestro tool of the Schrodinger suite (Schrödinger, 2019). Prepared mutant/controls structures were solvated using simple point charge, water molecule with 150 mM salt concentration ensuring the charge neutrality. Unrestrained MD simulations were carried out for 10 nanoseconds on each of the mutants on the residues lying within a 30 Å radius from the mutation site. Seven out of the 8 chains forming the IP region were represented as the receptor, while the chain containing the site of mutation is termed as the ligand and binding free energy (ΔG) value was calculated following a published protocol (Kotecha et al., 2015). The molecular mechanics/Poisson-Boltzmann surface area (MM-PBSA) module of the AMBER package was used for calculating the binding free energy (ΔG) value of all the mutations (Gohlke and Case, 2004; Genheden and Ryde, 2015) including the wild type and the negative control. The details of the mutations, their binding free energy values and S-ELISA reactivity were given in the **Table 1**.

**Table 1:** Description of mutants predicted by bioinformatics with binding free energy values (ΔG) of VLPs of FMDV serotype A/IND/40/2000 and their antigenic characterization using Sandwich ELISA.

Mutant^#^	Description of mutations in VP2, VP3, and IP region	Binding free energy (ΔG) (kcal/mol)^#^	S-ELISA reactivity (Absorbance)^$^
AM-1	H93Y (in VP2)	1153.52	1.94 ± 0.07
AM-2	V90F (in VP2)	1158.49	2.29 ± 0.12
**AM-3**	**double mutation: F62Y and H142D (F62Y in VP2 and H142D in VP3)**	**1091.92**	2.52 ± 0.06
AM-4	double mutation: V90T and H142N (VP2, VP3, and IP region)	1148.86	2.12 ± 0.15
AM-5	H93C (in VP2)	1161.90	2.34 ± 0.12
AM-6	H93F (in VP2)	1144.85	1.32 ± 0.07
AM-7	V90Q (in VP2)	1172.98	1.00 ± 0.03
AM-8	S97V (in VP2)	1178.55	0.61 ± 0.05
Wild type	Wild type (no mutation)	1201.16	2.42 ± 0.05
Negative control	F62N and H142R (VP2, VP3, and IP region)	1230.05	Not applicable
Inactivated FMDV serotype A antigen (positive control for S-ELISA)	Not applicable	Not applicable	2.65± 0.07

**^#^**The molecular mechanics/Poisson-Boltzmann surface area (MM-PBSA) module of the AMBER package was used for calculating the ΔG values from the dynamic trajectories of every mutant and A-WT structure. AM refers to mutations in either the VP2 and/or the VP3 protein of FMDV.

**^$^**Optical density or absorbance indicates the mean ± standard error (n=4) of the absorbance value at a wavelength of 492 nm measured in an ELISA reader. The positive control with inactivated antigen had an A_492_ absorbance of 2.65 ± 0.07, whereas the negative control Tn5 cell lysate had an A_492_ absorbance of 0.103 ± 0.05.The bolded value indicates the best mutant among all and therefore used in the further experiments in the research work.

### 2.4 Generation of recombinant baculovirus with mutations in the structural genes of FMDV

The predicted mutations (single or double) were introduced in the IP region (VP2-H3 helices and the proximal VP3) by PCR-based site-directed mutagenesis using KOD mutagenesis kit (Toyobo^©^, Japan) with wild type as control. The double mutants (AM-3 and AM-4) were constructed after confirming the presence of the first mutation by sequencing, and the recombinant plasmid containing the first mutation was used as template for second mutation. The resultant plasmid was confirmed to contain two mutations and was termed as the double mutants AM-3 and AM-4 (**Table 1**). The mutations were confirmed by sequencing and the plasmid was transformed into DH10 Bac™ *E. coli* competent cells (Thermo Fisher Scientific, USA). The recombinant clones (white colony) were selected, grown in LB broth and used for bacmid DNA isolation. The bacmid DNA was transfected into Sf-21 cells using Cellfectin (Thermo Fisher Scientific Inc., USA) to generate recombinant baculovirus for each mutant separately. The recombinant baculovirus was plaque purified and passaged in Sf-21 cells upto P_4_ to serve as stock virus.

### 2.5 Characterization of VLPs

The Tn5 cells were used for expression of 9 recombinant baculovirus VLPs. The expressed VLPs were harvested as described earlier (Terhuja et al., 2015) and characterized by sandwich ELISA (S-ELISA) (Bhat et al., 2013), electro-immunoblot transfer assay, transmission electron microscopy (TEM) (Ruiz et al., 2014) and immunofluorescence assay (Basagoudanavar et al., 2015).

### 2.6 Bulk production and quantification of VLPs

The recombinant P4 stock virus for each VLP was used at five multiplicities of infection (m.o.i) for bulk production in Tn5 cells by monolayer method and VLPs were harvested on 3-day post infection (dpi) when 80% cells showed cytopathic effect. The standard 146S antigen of FMDV serotype A was prepared as earlier (Barteling and Meloen, 1974) and used for VLP quantification in S-ELISA. S-ELISA was performed (Bhat et al., 2013) by diluting the 146S standard antigen (8 µg to 4 ng) and the VLPs by two-fold serial dilution. The absorbance (A492 nm) values of the standards were used for construction of standard curve using Curve expert 1.4 and VLPs were quantified.

### 2.7 Evaluating the thermostability of VLPs

VLPs were concentrated by ultracentrifugation in sucrose gradient (Ganji et al., 2018) and exposed to three different temperatures of 37, 45, and 56°C for different time points and the thermal stability was assessed by S-ELISA.

The initial concentration of 4 µg of each mutant and the wild type VLPs were stored at different temperatures of -20, 4, 24, and 37°C for 90 days. The stored VLPs were tested at 15 days intervals starting from day 0 to 90 post-storage (n=3/mutant/time point). The relative degradation of VLPs (%) tested in S-ELISA was calculated using the following formula:


$$Percent\;relative\;degradation=\left[\left(mean\;concentration\;of\;VLP\;at\;initial\;time\;point\right)-\left(mean\;concentration\;of\;VLP\;at\;particular\;time\;point\right)\right]/\left[mean\;concentration\;of\;VLP\;at\;initial\;time\;point\right]x100$$

### 2.8 Characterization of the unfolding temperature of VLPs

Differential scanning fluorescence (DSF) was performed to determine the unfolding temperature of VLPs using Protein binding fluorophore and SYPRO Orange dye in real-time PCR machine (Applied Biosystems real-time PCR machine 7500). Briefly, the temperature was ramped from 25 to 95°C with 1°C increments at an interval of 3 min in duplicate wells of a MicroAmp Optical 96-well plate. SYPRO orange fluorescence was produced with an excitation and emission wavelengths of 472 and 570 nm, respectively. The signal was detected using an ROX filter, and the minimum of the negative first derivative of the fluorescence curve was taken as the melting temperature (Tm). Each assay was performed pre and post exposing VLPs at 37, 45, and 56°C for 30 min and 60 min (n=3/mutant/time point).

### 2.9 Animal experimentation

Animal experiments were conducted as per the guidelines of Institutional Animal Ethics Committee (IAEC) of Indian Veterinary Research Institute (IVRI) campus, Hebbal, Bengaluru, India.

### 2.10 Determining the potency of stable VLPs by dose response study in guinea pigs

Dunkin Hartley guinea pigs were divided into five groups of six animals in each group and injected with VLP vaccine formulated with Montanide ISA 201VG adjuvant. Groups 1 to 5 were immunized (I/M) with 1, 2, 4, 8 and 12 µg/dose/animal, respectively. Animals were boosted on 30-day post vaccination (dpv) with respective antigen. On 58 dpv animals were challenged with 50 µL of serotype A 100 GPID_50_ virus (Median guinea pig infective dose_50_) by intradermally tracking in the left rear footpad (intra dermoplanter in the metatarsal pads). Further, animals were observed for ten days post challenge (dpc) for the appearance of lesions on the footpad. presence of lesions on any one of the non-inoculated foot pads indicates the animal is not protected, while absence of lesions in all the three non-inoculated footpads suggested that the animal is protected. To evaluate the antibody response against VLPs antigen, 2 mL of blood was collected intracardially on 0, 28 and 56 dpv and the sera were tested by Virus Neutralization Test (VNT) as per World Organisation for Animal Health (WOAH) (2022) protocols.

### 2.11 Comparison of potency of the AM-3 and wild-type VLPs in guinea pigs

Guinea pigs (n=44) were divided into four groups with eleven animals per group. Groups 1 to 4 were immunized intramuscularly at the left hind limb quadriceps muscle with PBS, 12 µg of AM-3 VLPs, 12 µg of wild type A-WT VLPs and 4 µg of FMDV A inactivated 146S antigen, respectively. Animals were boosted on 30 dpv with the respective antigens and challenged on 58 dpv with 100 GPID_50_ virus intra-dermally by tracking in the left rear footpad. Animals were observed for ten dpc for lesion scoring to determine percentage protection and potency of the VLPs. The antibody titres of the sera on 0, 28 and 56 dpv were carried out by VNT.

### 2.12 Detection of FMDV non-structural protein antibodies by 3AB NSP blocking ELISA

The 3AB NSP blocking ELISA was performed as reported (Hosamani et al., 2022). Briefly, the recombinant 3AB antigen was coated in 96 well MaxiSorp (Nunc, USA) ELISA plate and incubated for 60 min at 37°C. The guinea pig sera were diluted (1:1) in blocking buffer and added to the antigen-coated plate. Following washing with Tris (25 mM) buffered saline containing Tween-20 (0.025%), 10H9D8 mAb HRPO conjugate was added to the plate and incubated for 30 min at room temperature. Post washing, 50 µL TMB substrate was added and incubated for 15 min in dark at room temperature and the reaction was stopped by addition of 50 μL of 1.5 M H_2_SO_4._ The absorbance was measured at 450 nm and percentage inhibition (PI) was calculated using the following formula:


$$PI=\left[1-\left(OD_{TS}/OD_{NCS}\right)\right]\times 100$$

Where OD_TS_ is the mean OD of the test serum and OD_NCS_ is the mean OD of the negative control serum.

### 2.13 Statistical analysis

GraphPad Prism version 9.5.1 was used for statistical analysis. Data pertaining to degradation of VLPs and shelf-life at different temperature and time point, T_m_ values to find the thermal stability at high temperatures and neutralizing antibody titres of guinea pigs were analyzed by two-way ANOVA using Holm-Sidak *post-hoc* test. The dose to protection was modelled by simple binary logistic regression. Kaplan-Meier survival curve analysis was used for comparison of % inhibition of FMDV 3AB blocking ELISA with protection status among different vaccinated groups.

## 3 Results

### 3.1 Homology modelling and molecular dynamics of FMDV structural proteins

**Figure 1:**
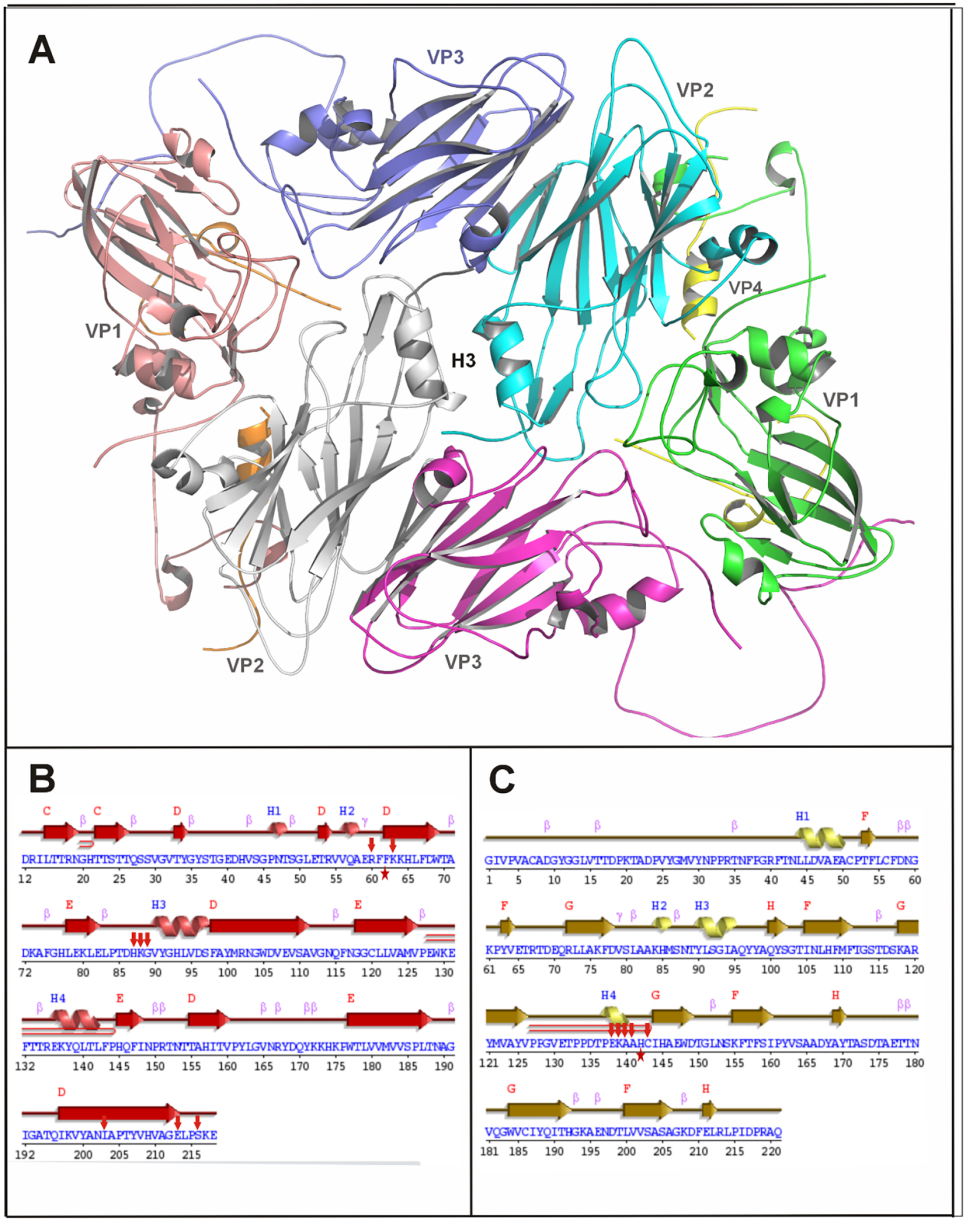
H3 helix region of VP2 forming a part of the inter-pentameric region: The crystal structure of the A22 Iraq strain (PDB code: 4GH4) was chosen as a template for constructing the homology model of FMDV serotype A/IND/40/2000. **(A)** Cartoon representation of the interpentameric region prepared using Schrodinger Maestro software. Residues interacting at the IP region are shown in the sticks. The IP region contains 8 peptide chains comprising 2 units each of VP1, VP2, VP3, and VP4. The amino acids V90, H93, S97 and F62 of VP2 and H142 of VP3 are targeted for mutation studies. Sequence and secondary structure features of VP2 **(B)** and VP3 **(C)**. The sites of mutation F62 in VP2 and H142 in VP3 are marked with red stars below the sequence. Red arrows indicate the residues that interact with the WT or mutant residue at the IP region.

**Figure 2:**
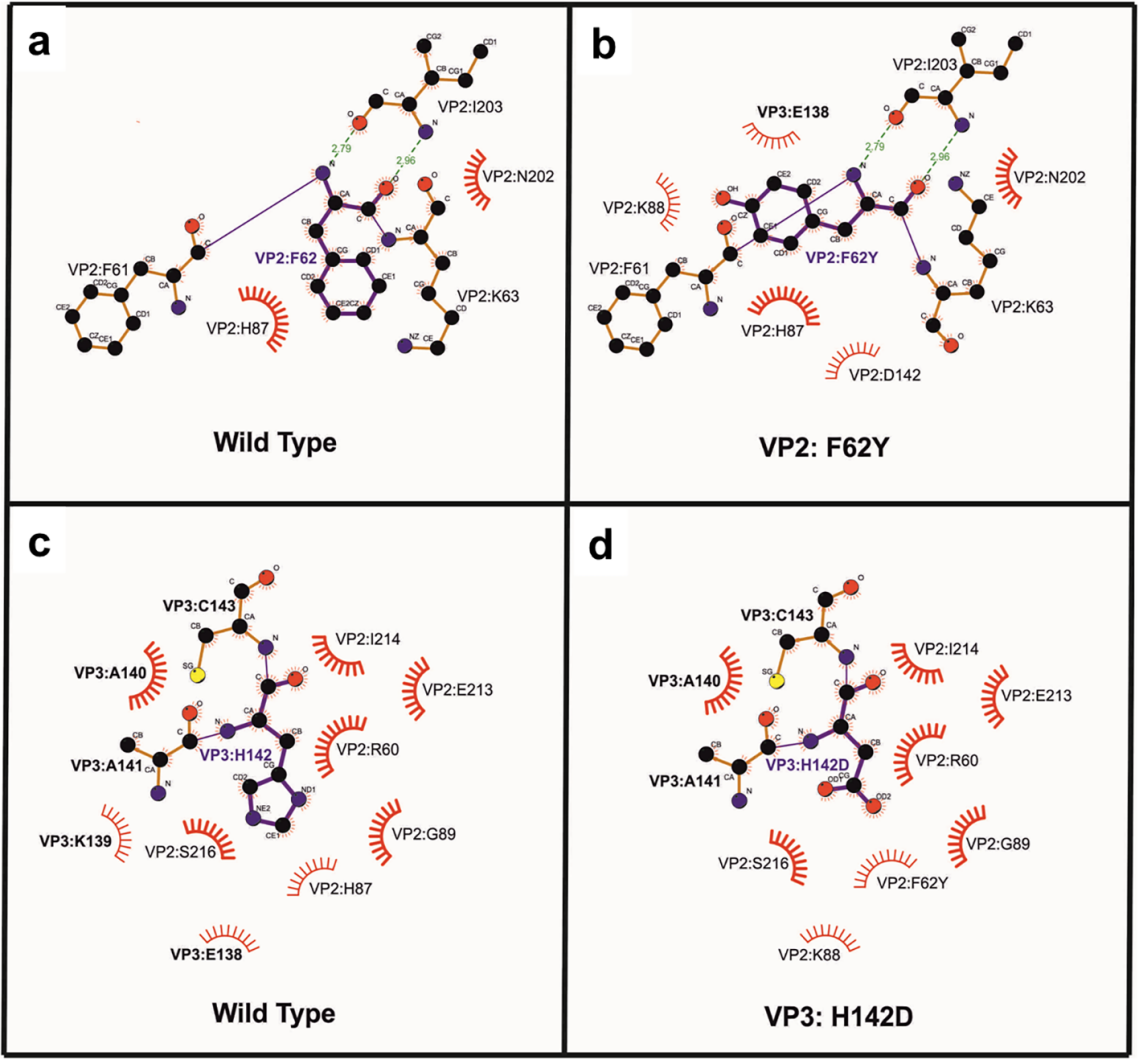
Schematic representation of the interactions in the double mutant, AM-3 of the FMDV serotype A determined using LigPlot: Panels **(a)** and **(b)** depict the changes in the intramolecular interaction due to mutation of F62Y in VP2. Similarly, panels **(c)** and **(d)** depict the same between wild-type and H142D in VP3. Arc with spikes indicate hydrophobic interactions. Substitution of F with Y in the VP2 region increased the hydrophobic interactions to five, of which two hydrophobic interactions involved E138 and D142 of the VP3 region of the neighboring pentamers. Substitution of H with D **(c)** resulted in reduced electrostatic repulsion in the VP3 region, which in turn provided the advantage of increasing the hydrophobicity by attracting Y62 and K88 of the VP2 region in the neighboring pentamer **(d)**.

Based on previous studies on other strains (Kotecha et al., 2015; Porta et al., 2013) and structure analysis at the IP region of the constructed model, F62, H93, S97, and V90 of VP2 and H142 of VP3 were selected for *in-silico* bioinformatic analysis (**Figure 1**). The differences in the local interactions due to mutations were analyzed by static and MD simulation trajectory models. In AM-3 double mutant (F62Y:H142D), two new interactions at the IP region were formed involving K88 of VP2 and E138 of VP3 at either end of the H3 helix (**Figure 2**; **Supplementary Figure 1**). Binding free energy (ΔG) of the serotype A wild type (A-WT) and mutants including controls were calculated (**Table 1**). The negative control was designed (F62N of VP2 and H142R of VP3) in such a way that it would disrupt the hydrophobic interactions of F62 and produce steric hindrance at the H142 site, thereby destabilizing the IP region. Of all the mutant structures analyzed by bioinformatics, AM-3 showed the lowest ΔG value (1091.9 kcal/mol), while A-WT (1201.1 kcal/mol) and the negative control mutant (F62N and H142R) showed higher ΔG values (1230.1 kcal/mol) as given in **Table 1**.

### 3.2 Characterization of recombinant baculovirus expressed VLPs

**Figure 3:**
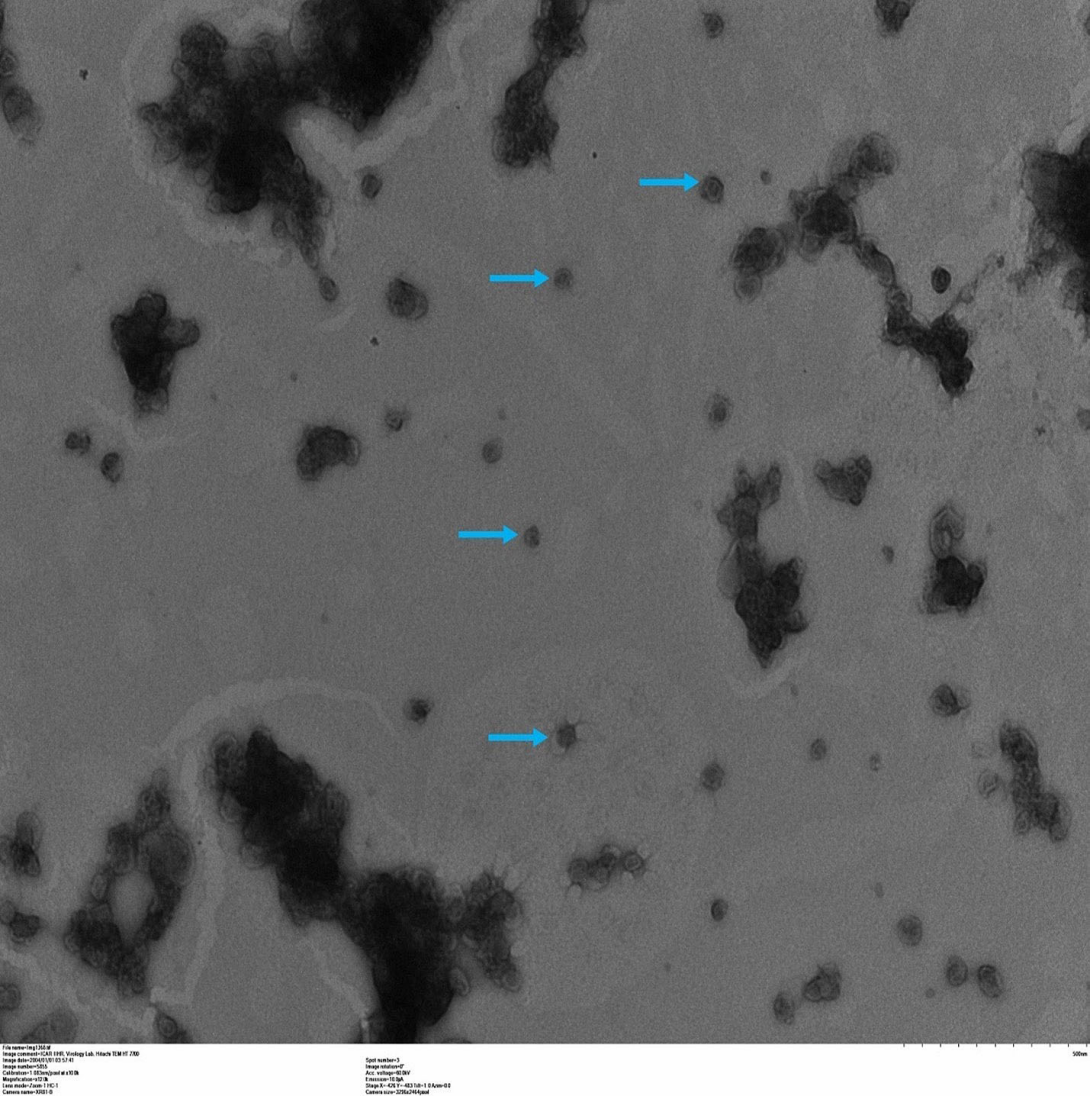
Demonstration of the transmission electron microscopic image of stable AM-3 virus-like particle: The AM-3 VLP was purified using different sucrose density gradients by ultracentrifugation. The fraction with peak reactivity was taken for TEM imaging. Samples were stained with 1% uranyl acetate for 1 min and dried at 37°C for 1h. Grids were examined under the transmission electron microscope (Tecnai, T12, USA) operated at 80 kV and images were acquired. The blue arrow indicates the AM-3 VLP in the size of 25–30 nm corresponding to an empty capsid.

Recombinant baculovirus containing the mutation in the VP2 and/or VP3 proteins were generated for eight predicted mutants and one wild type VLPs. They were characterized by S-ELISA and other techniques. The S-ELISA results showed that the optical density of the mutated VLPs was comparable with the wild type (**Table 1**). However, AM-3 showed the highest O.D of 2.52 than the other VLPs. The electro-immunoblot transfer assay demonstrated the expression of 25, 33 and 81 kDa corresponding to VP1/VP3, VP0, and P1-2A confirming the self-cleavage of polyprotein (P1) (**Supplementary Figures 2.1**, **2.2**). The ultracentrifuged fractions of S-ELISA showed fractions no. 14, 15 and 16 had the highest O.D values of above 2 as compared with other fractions (<1 O.D) (**Supplementary Figure 3**). The pooled and concentrated VLPs demonstrate the presence of 25–30 nm particles in the TEM images confirming the formation of intact AM-3 VLPs (**Figure 3**). The AM-3 VLPs reacted with a neutralizing mAb, which was previously characterized with 146S whole virus antigen, which confirmed the presence of similar antigenic epitopes in VLPs as that of whole virus. Further, the appearance of similar fluorescence in immunofluorescence test between the AM-3 and A-WT corroborated the intactness of the antigenic site (**Supplementary Figure 4**).

### 3.3 Quantification of expressed VLPs for stability and animal experiment studies

The 146S antigen was purified from the supernatant of FMDV serotype A infected BHK-21 cells by ultracentrifugation that showed a clear single white band (**Supplementary Figure 5**) with a concentration of 188 µg/mL and ratio of 259 nm/239 nm was 1.4 which indicates the purity of antigen. The concentration of VLPs was quantified based on the standard antigen O.D. values obtained in the S-ELISA, the standard curve was plotted using R^2^ value. We obtained maximum concentration of 0.1 to 0.2 µg/mL of mutant and wildtype VLPs in one 150 cm^2^ harvested flask.

### 3.4 Stability of the VLPs at different temperatures and time points

**Figure 4:**
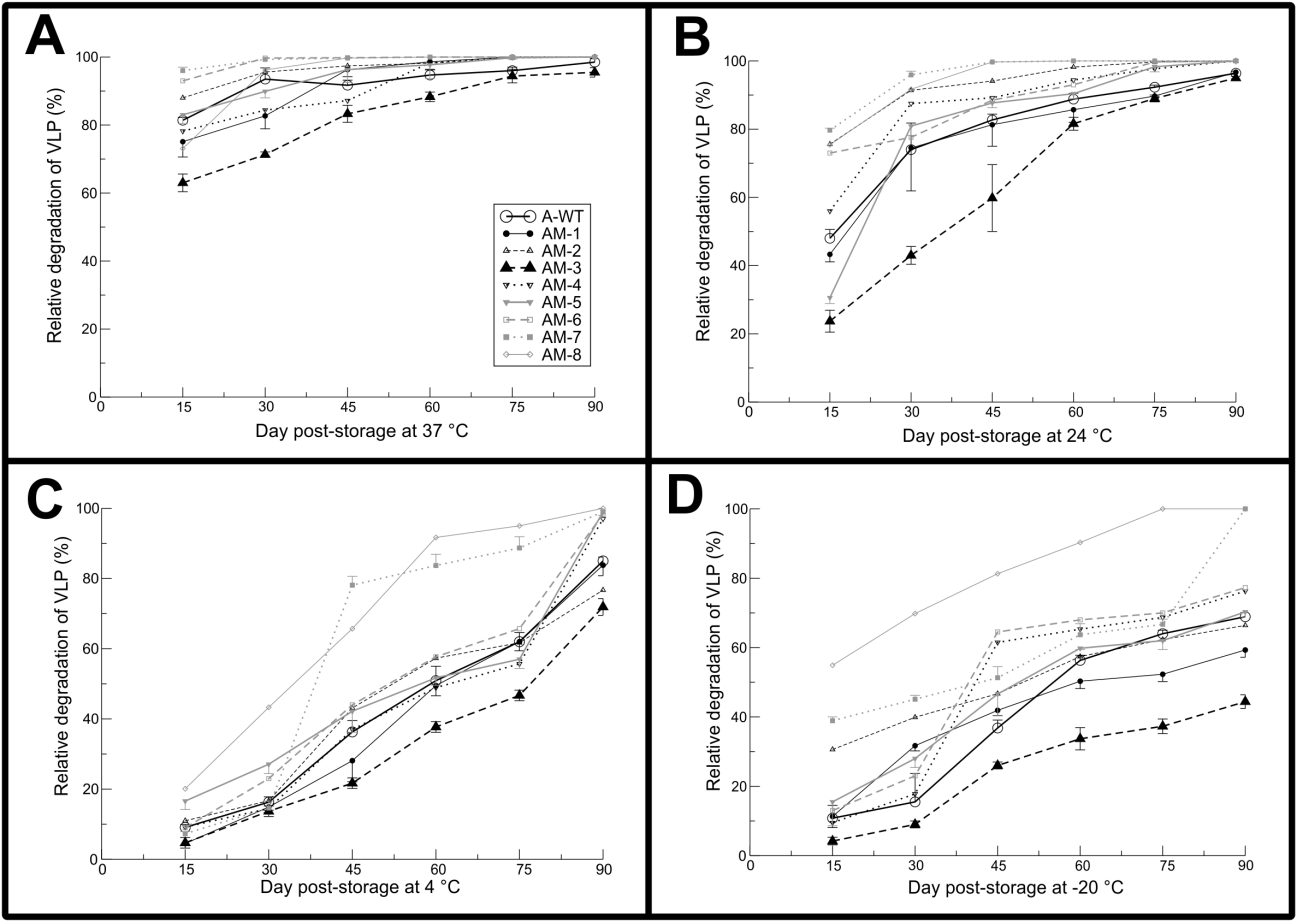
Stability of the mutant and wild type virus-like particles (VLPs) at different temperatures stored for 90 days: **(A-D)** plots indicate the relative degradation of VLPs stored at 37, 24, 4 and -20ºC. Antigen content on day 0 post-storage was considered 100%. Degradation (%) of the VLPs was calculated by considering the difference between the initial (Day 0 post-storage) antigen content *vis-à-vis* the day of testing. The aliquots of VLPs stored at different temperatures were subjected to S-ELISA at a 15-day interval from day 0 to 90 to determine the antigen stability by relative degradation (%). The data were analyzed by two-way ANOVA. Orthogonal contrast was done by comparing the A-WT with the mutants (AM). Holm-Siddak *post-hoc* test was used for comparing each mutant with the wild type. Significance was set at a 5% level. Each point in the line chart indicates mean ± standard error (n=3/time point). The mutant AM-3 showed significantly less degradation than A-WT at 37ºC on day 15 post-storage (P<0.01). The P values for the effect of the mutant, time and interaction were <0.001.

S-ELISA reactivity of mutants and wild type VLPs subjected to different temperatures and time intervals was shown in **Figure 4**. At 37°C (**Figure 4A**), AM-3 showed significantly lower degradation of 62.5% on 15 days post storage (dps) as compared to A-WT VLPs (>80% degradation) [95% CI: -27.65 to -10.65], AM-1 (P<0.05) and AM-8 (P<0.01) showed >70% degradation. On 30 dps, AM-3 showed a significantly lower degradation of <70% compared to A-WT (P<0.001) [95% CI: -29.85 to -12.85]. The stability was poor as evidenced by the antigen degradation of >90% after 60 dps for all the VLPs. At 24°C (**Figure 4B**), the degradation of the mutant VLP was in the range of 23 to 42.5%, and the difference was not statistically significant amongst all the VLPs, except A-WT VLPs which showed 47.5% antigen degradation on 15 dps (P>0.05) [95% CI: -38.90 to -10.10]. On 30 dps, AM-3 showed a significantly lower degradation of 42.5% in comparison with A-WT (72% degradation) (P<0.001) [95% CI: -43.92 to -15.12] and other VLPs (>90%). At 4°C (**Figure 4C**), the antigen degradation of mutant VLPs on 15, 30, 45, and 60 dps was comparable with that of A-WT (P>0.05); however, significantly lower degradation of 46% was recorded for AM-3 as compared to 62.5% for A-WT on 75 dps (P<0.001) [95% CI: -30.26 to -2.73]. At -20°C (**Figure 4D**), the antigenic degradation of mutant VLPs on 15, 30, 45, 60, and 75 dps was comparable with that of A-WT (P>0.05). However, AM-3 had a significantly lower degradation of 23.5% as compared to A-WT on 75 dps (P<0.001) [95% CI: -37.64 to -17.36].

**Figure 5:**
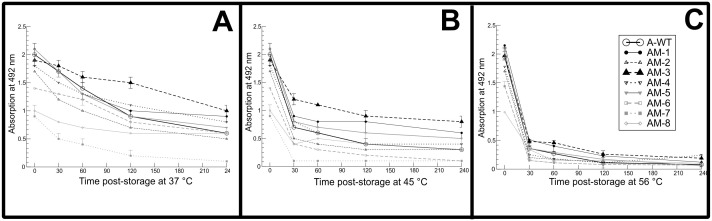
Effect of exposure to different high temperatures on the stability of mutant and A-WT VLPs: **(A–C)** plots show optical density values of VLPs exposed to 37, 45, and 56°C for 4 h. The VLPs were incubated at 37, 45 and 56ºC for 30, 60,120, and 240 min and the stability of VLPs was assessed by Absorbance (A_492_) values using S-ELISA. The data were analyzed by two-way ANOVA. Orthogonal contrast was done by comparing the A-WT with each mutant (AM1-8). Holm-Siddak *post-hoc* test was used for pair-wise comparison of the mean difference. Significance was set at a 5% level. Each point in the line chart indicates mean ± standard error (n=3/time point). The experiment was repeated thrice and result is presented. The P-values for the effect of the mutant, time, and interaction were <0.05.

The effect of exposure to high temperature on the stability of different VLPs is presented in **Figure 5**. At 37°C (**Figure 5A**), the reactivity of the mutant VLPs was comparable with that of A-WT for 0 to 120 min; however, at 240 min, it was significantly greater (0.46 OD) for AM-3 as compared to A-WT (P<0.05) [95% CI: -0.06 to -0.98]. At 45°C (**Figure 5B**), the reactivity of all the mutants was low and comparable with that of A-WT up to 240 min (P>0.05) except for AM-3, which had a significantly greater reactivity between 30 to 240 min (P<0.01) [95% CI: -0.04 to -1.05]. At 56°C (**Figure 5C**), no difference in the reactivity of mutant VLPs was observed as compared to A-WT at any time point (P>0.05) [95% CI: -0.31 to - 0.55].

**Figure 6:**
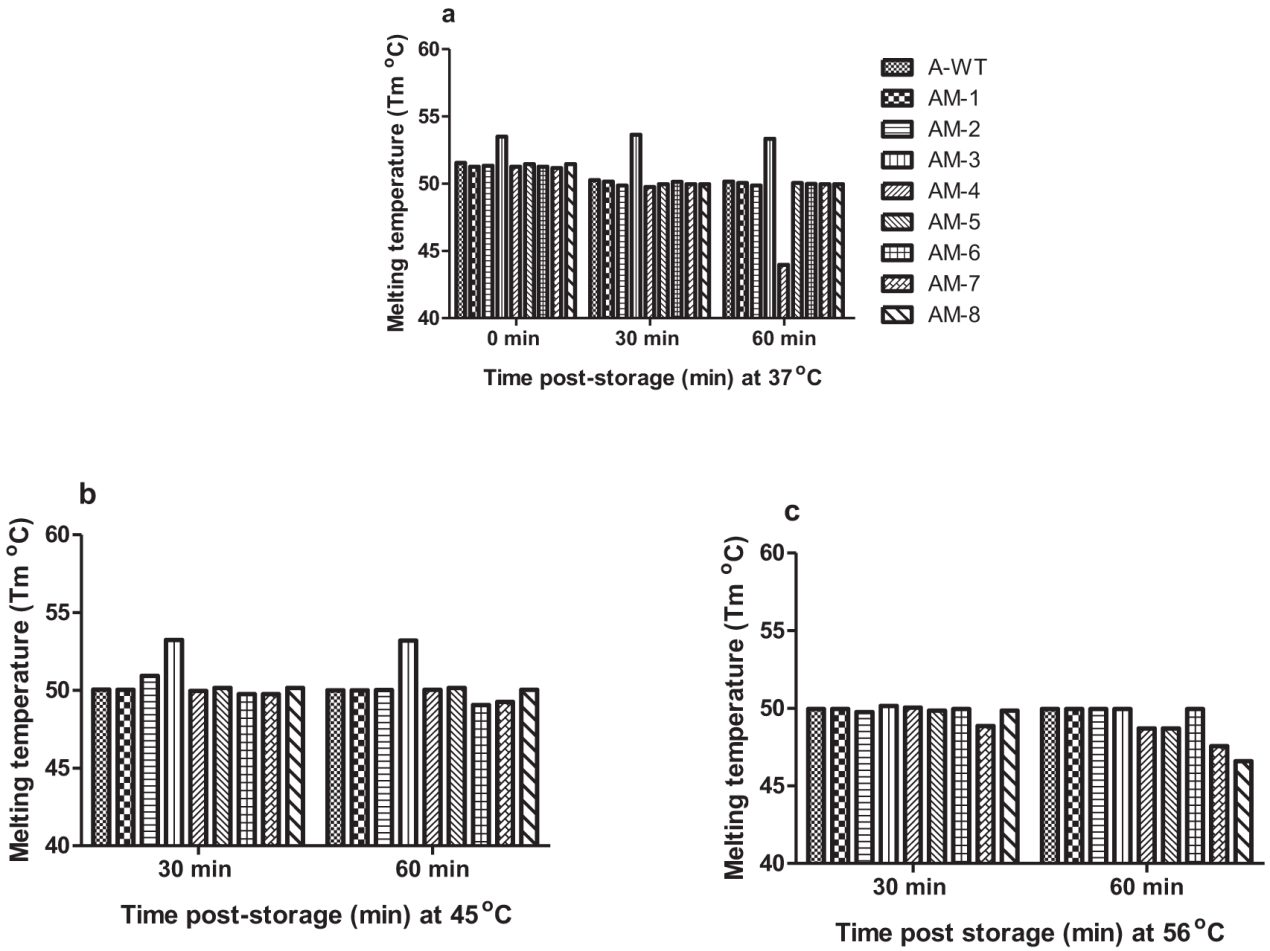
Differential scanning fluorescence (DSF) assay showing the melting temperature (Tm) curve of the VLPs of the FMDV serotype A: Thermostability of wild type and mutant VLPs were analyzed by DSF assay. The Tn5 cells expressed VLP were pelleted over 20% sucrose by ultracentrifugation at 1, 31,101 g for 5 h at 10°C. The concentrated VLPs were quantified by S-ELISA with known FMDV 146S standard antigen and 4 μg of VLPs each were used for the DSF assay. SYPRO orange fluorophore served as a signal at a concentration of 5X. Part **(a)** shows the Melt curve analysis showing the Tm of the VLPs before exposure to high temperature, at 37°C for 30 min and at 37°C for 60 min, Part **(b)** shows the Tm of the VLPs at 42°C for 30 min and at 42°C for 60 min. Part **(b)** shows the Tm of the VLPs at 56°C for 30 min and at 56°C for 60 min and Red, blue and black arrows indicate the Tm of VLP mutants, AM-3, AM-1 and A-WT, respectively. Exposure of VLPs to three different high temperatures for 60 min (n=3/mutant/time point) was analyzed by two-way ANOVA with orthogonal contrast using the Bonferroni *post-hoc* test. The melting temperature of the VLP mutants was comparable (P>0.05) when exposed to 37°C for 60 min; however, AM-3 demonstrated significantly high melting temperature at 45°C **(b)** and 56°C **(c)** as compared to A-WT at 30 min (P<0.001) [95% CI: 0.47 to 1.9] indicating thermostability.

The results of exposing the mutant VLPs to three different temperatures were studied using differential scanning fluorescence assay (**Figure 6**; **Supplementary Figure 6**; **Supplementary Table 2**). Before exposing VLPs to high temperatures, there was no significant difference in their melting temperatures (T_m_) and the negative control sample did not show any peak. At 37°C, there was no difference in the T_m_ of the mutant VLPs for 30 and 60 min (P>0.05) [95% CI: 2.58 to 3.65] incubation. However, when exposed to 45°C, the mean difference in the T_m_ (°C) of AM-3 and A-WT was 3.18 and 3.20 at 30 and 60 min, respectively (P<0.001) [95% CI: 3.01 to 4.22]. However, AM-3 demonstrated significantly high melting temperatures at 45°C and 56°C as compared to A-WT for 30 min (P<0.001) [95% CI: 0.47 to 1.9] incubation indicating a better thermostability. Based on the stability studies, it was observed that AM-3 VLPs are more stable compared to other VLPs.

### 3.5 Determination of protective efficacy of VLPs by dose response study in guinea pigs

**Figure 7:**
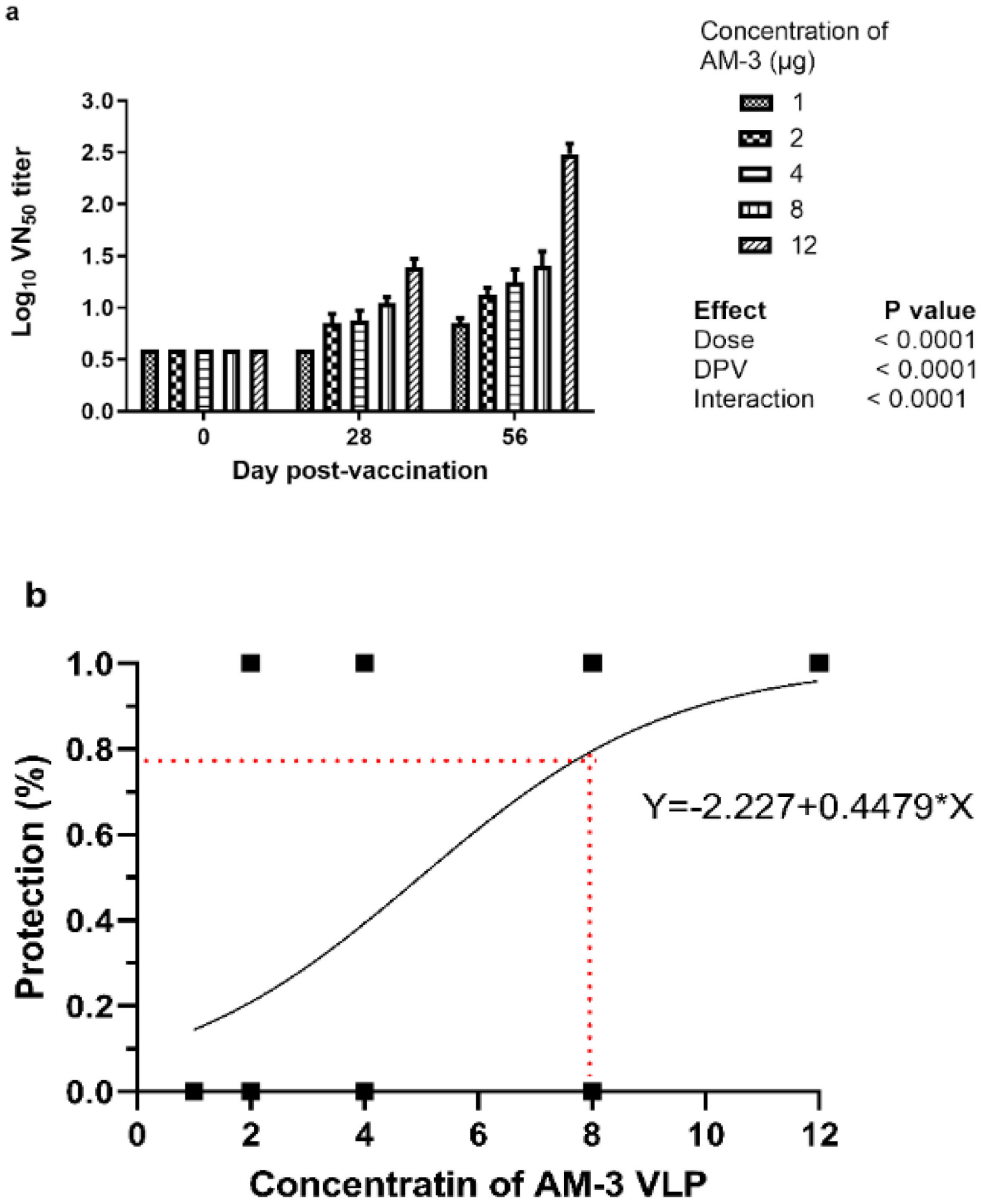
Dose response study of thermostable AM-3 VLPs in the guinea pig: A total of 36 seronegative guinea pigs were divided into six groups and six animals for each group. The PBS and thermostable AM-3 VLP at different doses such as (in µg), 1, 2, 4, 8 and 12 were used to immunize the 1, 2, 3, 4, 5 and 6 groups of guinea pigs, respectively. Serum was collected on days 28 and 56 post-vaccination to evaluate the vaccine-induced antibody titer. On day 58 post-vaccination all the animals were challenged with 50 µL of guinea pig adapted FMDV serotype A virus at 100GPID_50_ through intra-dermally by tracking in the left hind footpad. The absence of lesions on the non-inoculated foot pads on day 10 post-challenge was considered protected and the immunogenicity of AM-3 VLP was calculated. **(a)** Serum neutralization titer at each dpv was analyzed by two-way repeat measures ANOVA. **(b)** Simple binary logistic regression model was fit to find the log odds of protection (Y) using Graph Pad Prism 9.5.1. The results indicated that at thermostable AM-3 VLP at ≥ 8 µg conferred > 75% protection following challenge.

On the basis of the results of the stability experiments, stable AM-3 and wild-type VLPs were chosen for further animal experimentation. The serum neutralizing antibody titres of the immunized guinea pigs were presented in **Figure 7a** as a log_10_ value and **Table 2** represents the rise in serum neutralizing antibodies post immunization. The mean log_10_ titres of group 1–5 values were calculated as 0.85, 1.13, 1.25, 1.40 and 2.48, respectively. The dose to protection was modelled by simple binary logistic regression and the results indicated that AM-3 VLP at 12 µg/dose conferred complete protection following challenge (**Figure 7b**; **Supplementary Figure 7**).

**Table 2:** Neutralizing antibody titers of sera from immunized guinea pigs and their NSP-blocking ELISA results in comparison with protection against virus challenge.

Group	Guinea pig ID	Antibody titer on 0 dpv	Antibody titer on 28 dpv titer	Antibody titer on 56 dpv titer	Protection status (based on lesion scoring)	Percentage protection (%)	PI Value (%)^#^ NSP blocking ELISA	Protection status (based on NSP blocking ELISA) *
Group I PBS administered	657	< 0.60	< 0.60	< 0.60	NP	0	96	NP
	372	< 0.60	< 0.60	< 0.60	NP		95	NP
	303	< 0.60	< 0.60	< 0.60	NP		94	NP
	533	< 0.60	< 0.60	< 0.60	NP		94	NP
	297	< 0.60	< 0.60	< 0.60	NP		95	NP
	530	< 0.60	< 0.60	< 0.60	NP		96	NP
	777	< 0.60	< 0.60	< 0.60	NP		97	NP
	536	< 0.60	< 0.60	< 0.60	NP		98	NP
	540	< 0.60	< 0.60	< 0.60	NP		95	NP
	538	< 0.60	< 0.60	< 0.60	NP		98	NP
	539	< 0.60	< 0.60	< 0.60	NP		95	NP
Group II AM-3 administered	995	< 0.60	1.20	2.26	P	90.91	3	P
	372	< 0.60	0.90	1.95	P		12	P
	303	< 0.60	1.36	2.41	P		7	P
	505	< 0.60	0.90	2.11	P		10	P
	508	< 0.60	0.90	1.20	NP		88	NP
	61	< 0.60	1.36	2.26	P		5	P
	10	< 0.60	1.65	2.26	P		4	P
	529	< 0.60	0.90	2.11	P		8	P
	532	< 0.60	1.04	2.56	P		5	P
	910	< 0.60	1.51	2.71	P		8	P
	657	< 0.60	1.65	2.41	P		10	P
Group III A-WT administered	943	< 0.60	0.90	0.90	NP	36.36	92	NP
	759	< 0.60	0.90	0.90	NP		94	NP
	610	< 0.60	0.90	1.04	NP		95	NP
	536	< 0.60	1.20	1.81	P		17	P
	121	< 0.60	0.90	2.71	P		12	P
	410	< 0.60	1.04	1.04	NP		96	NP
	38	< 0.60	0.90	1.20	NP		97	NP
	730	< 0.60	0.90	1.04	NP		94	NP
	529	< 0.60	0.90	1.04	NP		95	NP
	346	< 0.60	1.20	1.95	P		11	P
	556	< 0.60	1.04	1.51	P	100	9	P
Group IV Inactivated serotype A 146S antigen administered	930	< 0.60	1.20	2.26	P		20	P
	101	< 0.60	1.36	1.95	P		35	P
	741	< 0.60	1.04	2.41	P		18	P
	346	< 0.60	0.90	2.11	P		23	P
	844	< 0.60	1.04	2.26	P		37	P
	821	< 0.60	1.51	3.01	P		29	P
	NI	< 0.60	0.90	2.11	P		32	P
	196	< 0.60	0.90	2.26	P		36	P
	958	< 0.60	1.36	2.56	P		28	P
	760	< 0.60	1.20	3.01	P		31	P
	551	< 0.60	1.51	3.01	P		34	P

Antibody titers are expressed as log_10_ values of VNT for all serum samples. The VNT values of more than 1.2 log_10_ in the serum samples at 56 dpv of guinea pigs were 100% correlated with protection against virus challenge.

^#^Results of percentage inhibition (PI) in NSP blocking ELISA. A PI value of less than 40% indicated that the animal was protected against challenge and that there was no virus multiplication, whereas a PI value of more than 40% suggested that the challenge virus multiplied in guinea pigs, produced antibodies against nonstructural proteins (NSP) and was not protected against challenge by lesion scoring.

*Indicates that the guinea pig is protected against challenge on the basis of the PI values of NSP blocking ELISA, which correlates 100% with the clinical protection based on lesion scoring given in the “Protected status (lesion scoring)” column.

### 3.6 Immunogenicity studies of AM-3 and A-WT VLPs in guinea pigs

**Figure 8:**
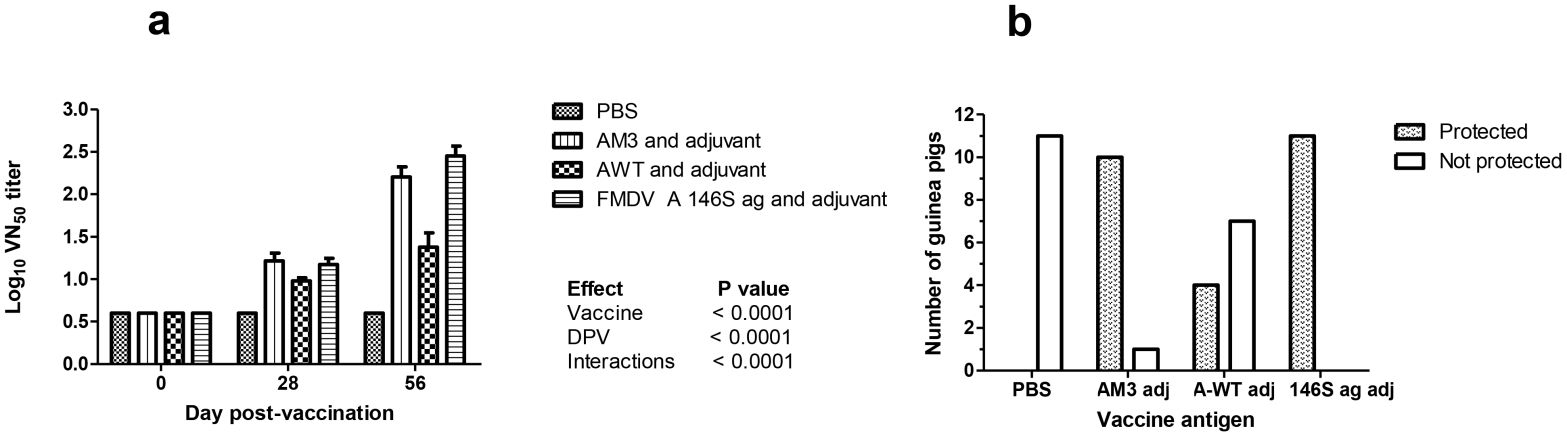
Immunogenicity of thermostable AM-3 VLPs in guinea pigs: A total of 44 seronegative guinea pigs were divided into four groups and eleven animals for each group. The PBS, 12 µg of AM-3 VLPs, 12 µg of wild type A-WT VLPs and 4 µg of FMDV serotype A 146S inactivated antigen were used to immunize the 1, 2, 3 and 4 groups of guinea pigs, respectively. All the antigens were given at the intramuscular site of the left hind limb quadriceps muscle. Blood was collected on days 28 and 56 post-vaccination. Booster was given on day 30 post-vaccination with the same antigen concentration and volume. On day 58 post-vaccination all the guinea pigs were challenged with 50 µL of guinea pig-adapted FMDV serotype A virus at 100GPID_50_. The absence of lesions on the secondary foot pads on day 10 post-challenge was considered as protected and the immunogenicity of vaccine antigens was calculated. Graphs a and b indicate the serum neutralization titer value of guinea pigs in each group and the number of protected and non-protected guinea pigs in each group, respectively.

The antibody titres (log_10_) of guinea pigs tested by VNT is shown in **Figure 8a** and **Table 2**. On 56 dpv, mean antibody titre was log_10_ of 0.60, 2.32, 1.85 and 2.62 for groups one, two, three and four, respectively. The lesion scoring and percentage protection in vaccinated guinea pigs were calculated as shown in **Supplementary Figure 7**. The mean antibody titre of the guinea pigs vaccinated with AM-3 VLPs was comparable with the inactivated FMDV antigen group. Upon challenge, the numbers of protected animals were 0, 10, 4 and 11 in the group of one, two, three and four, respectively as shown in **Figure 8b** indicating that FMDV neutralizing antibodies were elicited by thermostable VLPs.

### 3.7 Detection of 3AB antibodies of FMDV by NSP blocking ELISA

3AB NSP blocking ELISA with >40% Percentage Inhibition (PI) in challenged guinea pigs indicated that the animal was not protected by vaccination, which allowed the challenge virus to replicate and produce NSP antibodies and the results were given in **Table 2** and **Supplementary Figure 8**. All the guinea pigs in the negative control group administered with PBS (Group 1) showed >90% PI values which indicated that all the guinea pigs were not protected against challenge and showed lesions in all the four legs suggesting that the NSP antibodies are >40% due to multiplication of challenge virus used in the experiment in the guinea pigs. In group 2, guinea pigs administered with AM-3 VLPs, out of 11, only 1 guinea pig showed PI values of 88% in NSP blocking ELISA which is >40% of cut-off value and animal was not protected against challenge and thus correlating with protection data. In group 3, out of 11, 7 animals showed PI values of >40% (94% for GP 759; 95% for GP 610; 96% for GP 410; 97% for GP 38; 94% for GP 230 and 95% for GP 529) were not protected against challenge. Similarly in group 4, out of 11, all 11 guinea pigs did not show <40% PI values and all got protected against challenge. These data were further correlated with the antibody titres measured by VNT on 56 dpv which is more than protective titre of 1.2 log_10_ as shown in **Table 2**. Kaplan-Meier survival curve analysis indicated that the thermostable AM-3 antigen administered group had 95% protection and was comparable with that of 146S administered positive control with 100% protection (**Table 2**; **Supplementary Figure 8**).

## 4 Discussion

FMD is a highly contagious and economically devastating disease affecting cloven-footed animals. Recently, we generated acid-stable VLPs for the FMDV serotype O and Asia1 by constructing a substitution mutant N17D in VP1 and the mutant VLPs were tested by S-ELISA and TEM suggesting the intactness of the mutant VLPs (Deepak et al., 2019). Here, we report the successful engineering of thermostable VLPs by stabilizing the IP region based on the *in-vitro* thermal stability studies. There was a single report of thermostable H93C mutant in VP2 of FMDV serotype A22 Iraq strain, which targeted the IP region (Porta et al., 2013) and this is the first report of generation of thermostable VLPs of FMDV serotype A/IND/40/2000 (Indian vaccine strain) in both *in-vitro* laboratory test and *in-vivo* guinea pig challenge studies. The *in-silico* strategy was based on an earlier report (Kotecha et al., 2015) in which mutation of a thermostable candidate targeting the IP regions of O1/Manisa/Turkey/69 (O1M), and SAT2/ZIM/7/83 (SAT2) was made using crystallographic structures of earlier templates using homology modelling. It was observed that the templates, O1M and SAT2, share sequence similarities of 98% and 78%, respectively. We used the crystal structure of the A22 Iraq strain (PDB code: 4GH4) for homology modelling (**Figure 1**) since no crystallographic data is available for A/IND/40/2000. The sequence identity between the capsid coding regions of the two strains was 88% which justified the choice of our template. However, the stretches of interpentameric amino acids are dissimilar between two strains. Hence the thermostable amino acid responsible for A22 Iraq strain may not be the same for A/IND/40/2000 strain as hypothesized. First, we identified the thermolabile amino acids by taking inputs from previous studies (Kotecha et al., 2015; Porta et al., 2013) (**Figure 1**).

Histidine is a basic amino acid, hence the presence of a couple of them at the IP interface will be a disadvantage in terms of its stability. We tried mutating H to either F or Y, thus retaining the ring structure and thereby removing electrostatic instability. H93Y in VP2 can be anticipated to make a stabilizing hydrogen bonding interaction while H93F in VP2 could give rise to aromatic interactions. The other notable residues that could potentially interact at the H3 region are S97 or V90, as they lie near each other across the H3 helix region. Further S97V, V90F, V90T, and V90Q in IP region were constructed, and their respective thermal stabilities were studied. Though the IP region is mainly targeted for conferring thermostability (Kotecha et al., 2015; Porta et al., 2013; Ganji et al., 2018), we also considered H142 of VP3 which lies in close proximity to the H3 helices interface (**Figure 1**) region as it is essential for uncoating of the viral genome of FMDV (Ellard et al., 1999).

H142 of VP3 is located at the C terminus of a helix and is in proximity to R60 and F62 of VP2. Therefore, this H142 was mutated to D to introduce favourable electrostatic interactions with R60. Also, to increase the polar interaction at this juncture, F62 of VP2 was mutated to Y. Thus, a double mutant F62Y: H142D (AM-3) was constructed outside the IP interface adjacent to the H3 region. F62Y showed a change in the side-chain conformation, forming an additional non-covalent interaction with E138 in VP3 and K88 in VP2. H142D also stabilized electrostatic interactions with K88 in VP2 (**Figure 2**; **Supplementary Figure 1**) and the additional hydrogen bonds being formed with capsid of the AM-3 VLPs are based on *in-silico* bioinformatics predictions. Thus, this novel approach of designing mutants outside the H3 region proved fruitful. As a negative control, a destabilizing double mutant was constructed with F62N in VP2 and H142R in VP3 which resulted in a loss of hydrophobic interactions whereas H142R introduces a bulky group. Of all the mutants, AM-3 was the best candidate as revealed by the computational low binding free energy value of 1091.92 kcal/mol. Further, *in-vitro* and *in-vivo* studies were taken up to validate the computational findings.

The mutant VLPs produced in Tn5 insect cells were characterized by S-ELISA, electro-immunoblot transfer assay, transmission electron microscopy and immunofluorescence assay. High reactivity in S-ELISA suggested they were serotype-specific and little cross-reactivity with other serotypes in comparison to blank control. The mock-infected Tn5 cell lysates failed to react in serotype-specific S-ELISA (A492nm <0.1) indicating that the expressed VLPs possessed antigenic epitopes similar to native virus, which is in agreement with the previous report (Bhat et al., 2013). Further, demonstrations of 25, 33 and 81 kDa bands corresponding to VP1/VP3, VP0 and P1-2A, respectively in electro-immunoblot transfer assay with the mutant VLPs confirmed the presence of structural proteins required for the assembly of VLPs as reported earlier (Liang et al., 2014). The utility of AM-3 VLPs as a vaccine candidate essentially relies on the conservation of antigenic epitopes similar to that of the parental virus which was evident from the transmission electron micrograph, 25–30 nm size virus particles demonstrated the formation of the intact VLPs as empty capsids of FMDV A serotype and it ensured the post translational 2A protein self-cleavage process (**Figure 3**) (Deepak et al., 2019). The mAb used for VLPs characterization in immunofluorescence specifically binds to the 146S antigenic site 1 on the VP1 region of the whole virion in S-ELISA. The results supported that the mutated VLPs retained the epitopes similar to the parental virus (**Supplementary Figure 4**) and it confirms the intactness of the capsids expressed in insect cells.

The mutant VLPs when exposed to shelf-life studies by storing at 37°C for 90 days, AM-3 showed a relative degradation which was the least among all VLPs (P<0.001) [95% CI: -29.85 to -12.85] (**Figure 4**). The accelerated thermal stability study was evaluated by exposing the VLPs to three different high temperatures based on an earlier report on FMDV VLPs serotype O (Ganji et al., 2018). The mutant AM-3 had shown a significantly higher reactivity at 37°C for 240 min, whereas the antigen reactivity of A-WT and other mutants VLPs had shown significant decrease in reactivity <1.0 (P<0.05) [95% CI: -0.06 to -0.98] (**Figure 5**) indicating the intactness of AM-3. It is reported that mutant antigen derived out of the infectious clone of FMDV serotype O1/Manisa/Turkey/69 strain maintained the structural integrity (Kotecha et al., 2015) when exposed to 37°C for 96h. A similar trend in the A_492_ value of the mutant VLPs was observed at 45°C. We choose a Tm range of 45 to 56°C as it dissociates the empty capsid (146S) into pentamers (12S) and protomers (5S) as reported earlier (Kotecha et al., 2015). The accelerated thermal stability assessed by DSF assay revealed that AM-3 showed a significantly higher T_m_ at 45°C (P<0.001 [95% CI: 3.01 to 4.22]; **Figure 6**; **Supplementary Table 2**) which encouraged our findings of thermal stability. From the *in-silico* and *in-vitro* studies, AM-3 had shown increased thermal stability compared with other mutant and wild type VLPs. Hence, AM-3 and wild type VLPs were considered for *in- vivo* experiments.

Guinea pigs were used as model for immunogenicity studies of new generation FMD vaccine (Bhat et al., 2013; Terhuja et al., 2015). Guinea pig based FMD vaccine testing is optimized as an alternate for cattle serology-based potency testing. Hence, guinea pigs are widely used for the initial characterization of the antigenic properties of FMD vaccine candidates. The FMDV Asia1 empty capsids/VLPs induce neutralizing antibodies in the guinea pigs, but their levels were lower than those generated by the commercial vaccine because of quantity of the VLPs might have been lower than in the conventional inactivated vaccine (Cao et al., 2009). Guinea pig studies with Asia1 serotype VLPs designed using bioinformatics showed comparable immunogenicity with that of whole virus vaccine (Aparna et al., 2024). To overcome the issue of VLP dosage, a dose–response study was conducted with different concentrations. The results indicate that (1 PD_50_ is 3.81 [95% CI: 2.3 - 6.33]) for making a 1 PD_50_ vaccine, 3.81 µg of antigen should be present in the vaccine. Hence, to develop 3 GPPD_50_ vaccines, 11.43 µg of AM-3 VLPs are needed.

The neutralizing antibody titre of AM-3 VLPs vaccinated group was comparable with the FMDV inactivated antigen group, while the A-WT VLPs group showed low neutralizing antibody titre as compared to AM-3 and inactivated antigen groups. As a result of the immunogenicity study, significant differences in antibody titres were observed (P<0.001) [95% CI: 1.26 to 1.94] (**Figure 8**) among the different groups. On 3 dpc, lesions were observed in the PBS group similar to the observations found in a previous study (Cao et al., 2009) and they reported the lesions were observed on 2 dpc onwards. However, on 10 dpc, the percentage of protected animals in groups 1 to 4 is 0, 90, 36 and 100%, respectively. The value in range of 95% confidence interval of groups one, two, three and four are 0 - 22%, 62 - 98%, 15 - 65% and 78 - 100%, respectively.

This study demonstrated that capsid stability can be enhanced without compromising immunogenicity, and this might be a rational strategy for improving vaccine efficacy (Porta et al., 2013). We determined the protection status of the guinea pig against the challenge virus by NSP blocking ELISA, showing 3AB antibody were produced in infected animals but not in VLPs and inactivated antigen groups. In group 1, 2, 3 and 4, out of 11 animals, 0, 10, 4 and 11 animals were protected against challenge, respectively showing NSP antibodies with PI value <40%. Similarly, out of 11 animals, 11, 1, 7 and 0 guinea pigs were not protected against challenge, respectively indicating NSP antibodies PI value >40% and these results correlated well with the titres of VNT with cut-off of 1.2 log_10_ as protective titre in guinea pigs. We are the first to assess the guinea pig protection or non-protection status by use of NSP blocking ELISA apart from the clinical lesion scoring after live virus challenge. Hence, it is evident from the ELISA results that stable VLPs can be applied for DIVA purposes.

Here we demonstrated a proof of concept for producing a thermostable FMDV serotype A VLPs of the Indian vaccine strain. Most importantly, to employ the DIVA strategy which is eventually helpful in differentiating infected from vaccinated animal in seromonitoring under FMD control programme. These results encourage further work towards the VLPs comprising either monovalent or multivalent thermostable antigens, which can be produced according to the requirements for serological surveillance as novel combined thermostable VLPs vaccines. However, the study has certain limitations such as concentration of VLPs vaccine candidate should be quantified by mAb based ELISA before going to the natural host and importantly cell mediated immune response should be explored since P1 sequence contains both B-lymphocyte and T-lymphocyte epitopes for assessing the long-term immunity. Collectively, our results suggested that AM-3 withstood dissociation against protein monomers (Zepeda-Cervantes et al., 2020) and is a potential candidate for developing a thermostable VLP vaccine or diagnostic for FMD. Guinea pig immunogenicity study indicated that VLPs elicited sufficient immune response to overcome the virus infection, making them a potential agent for next generation-based vaccines.

Although the VLPs used in this study holds a potential to induce optimal immune response with respect to studies conducted in guinea pigs, a finer detail would be required to conclude the fact that VLPs do hold the vaccinal response in the definitive hosts. Hence, in this study, the results depict that preliminary studies unveil the immune response of VLPs only in the guinea pigs. Futuristic approach will involve experimentation on natural hosts with detailed and accurate evaluation of cell-mediated immune responses.

## 5 Conclusion

In the present study, bioinformatic approach was successfully employed to determine the amino acids involved in the stabilization of capsids/VLPs of FMDV serotype A/IND/40/2000. The mutated VLPs were assessed for their thermal stability by *in-vitro* testing and their immunogenic potential in guinea pig was confirmed by virulent virus challenge which could be used as a candidate vaccine with DIVA assay for effective FMD control and eradication in endemic settings. Thus, our results suggested a significant contribution in developing a thermostable VLP based vaccine which needs to be tested in ruminants like cattle, sheep, goat and buffaloes for future use in animal vaccination programme.

## Data Availability

The raw data supporting the conclusions of this article will be made available by the authors, without undue reservation.
