# Supplementary material for: Bioinformatics based design of thermostable virus-like particles-based vaccine for foot-and-mouth disease serotype A and in-vivo evaluation in guinea pigs
Source: Front Cell Infect Microbiol. 2026 Mar 30;16:1760751. doi: 10.3389/fcimb.2026.1760751 (PMC13071054; doi:10.3389/fcimb.2026.1760751)
Supplement: Supplementary file 1 [file Table1.docx]

**Supplementary Table 1. Sequences of the primers used in PCR for amplification of P1-2A and 3C regions of FMDV serotype A/IND/40/2000**

| **Name** | **Sequence (5’ – 3’)** | **Nucleotides** | **Nucleotide binding position** |
| --- | --- | --- | --- |
| BamHI VP4 F | 5’- TGG GAT CCA TGG GAG CTG GGC AAT CCA GTC -3’ | 30 | VP4 region |
| SpeI 2B29 R | 5’- TGA CTA GTC TCC ACC AGY TTG GAG AAG T -3’ | 28 | VP2 region |
| SpeI 3B3C F | 5’-TGA CTA GTG CTA AGA ACC TGA TTG TCA CTG AGA GTG GTG CCC CRC -3’ | 45 | 3C region |
| O3C HindIII R | 5’- CCC AAG CTT CTC ATG GTG TGG TAC GGG ATC -3’ | 30 | 3C region |

**Supplementary Table 2. Melting temperature (T_m_) of mutant VLPs of FMDV serotype A exposed to 37ºC, 45ºC, and 56ºC for 30 and 60 min determined by Differential scanning fluorescence (DSF)**

| **Mutant** | **T_m_ (ºC) of VLP at different time post-storage (min) exposed to 37ºC** | | | **T_m_ (ºC) of VLP at different time post-storage (min) exposed to 45ºC** | | **T_m_ (ºC) of VLP at different time post-storage (min) exposed to 56ºC** | |
| --- | --- | --- | --- | --- | --- | --- | --- |
|  | **0 min** | **30 min** | **60 min** | **30 min** | **60 min** | **30 min** | **60 min** |
| AM-1 | 51.25 | 50.16 | 50.06 | 50.04 | 50.00 | 49.96 | 49.96 |
| AM-2 | 51.35 | 49.86 | 49.86 | 50.94 | 50.02 | 49.76 | 49.96 |
| AM-3 | **53.50** | **53.64** | **53.34** | **53.24** | **53.20** | **50.16** | **49.96** |
| AM-4 | 51.25 | 49.76 | 43.96 | 49.96 | 50.04 | 50.06 | 48.70 |
| AM-5 | 51.45 | 49.96 | 50.06 | 50.16 | 50.16 | 49.86 | 48.70 |
| AM-6 | 51.25 | 50.14 | 49.98 | 49.76 | 49.06 | 49.96 | 49.96 |
| AM-7 | 51.15 | 49.96 | 49.96 | 49.76 | 49.26 | 48.86 | 47.58 |
| AM-8 | 51.45 | 49.96 | 49.96 | 50.16 | 50.04 | 49.86 | 46.60 |
| A-WT | 51.55 | 50.26 | 50.16 | 50.06 | 50.00 | 49.96 | 49.96 |
| Negative control | No regular peak | | | | | | |
