# Supplementary material for: Bioinformatics based design of thermostable virus-like particles-based vaccine for foot-and-mouth disease serotype A and in-vivo evaluation in guinea pigs
Source: Front Cell Infect Microbiol. 2026 Mar 30;16:1760751. doi: 10.3389/fcimb.2026.1760751 (PMC13071054; doi:10.3389/fcimb.2026.1760751)
Supplement: Supplementary file 2 [file Table2.docx]

**Supplementary Figures**

**
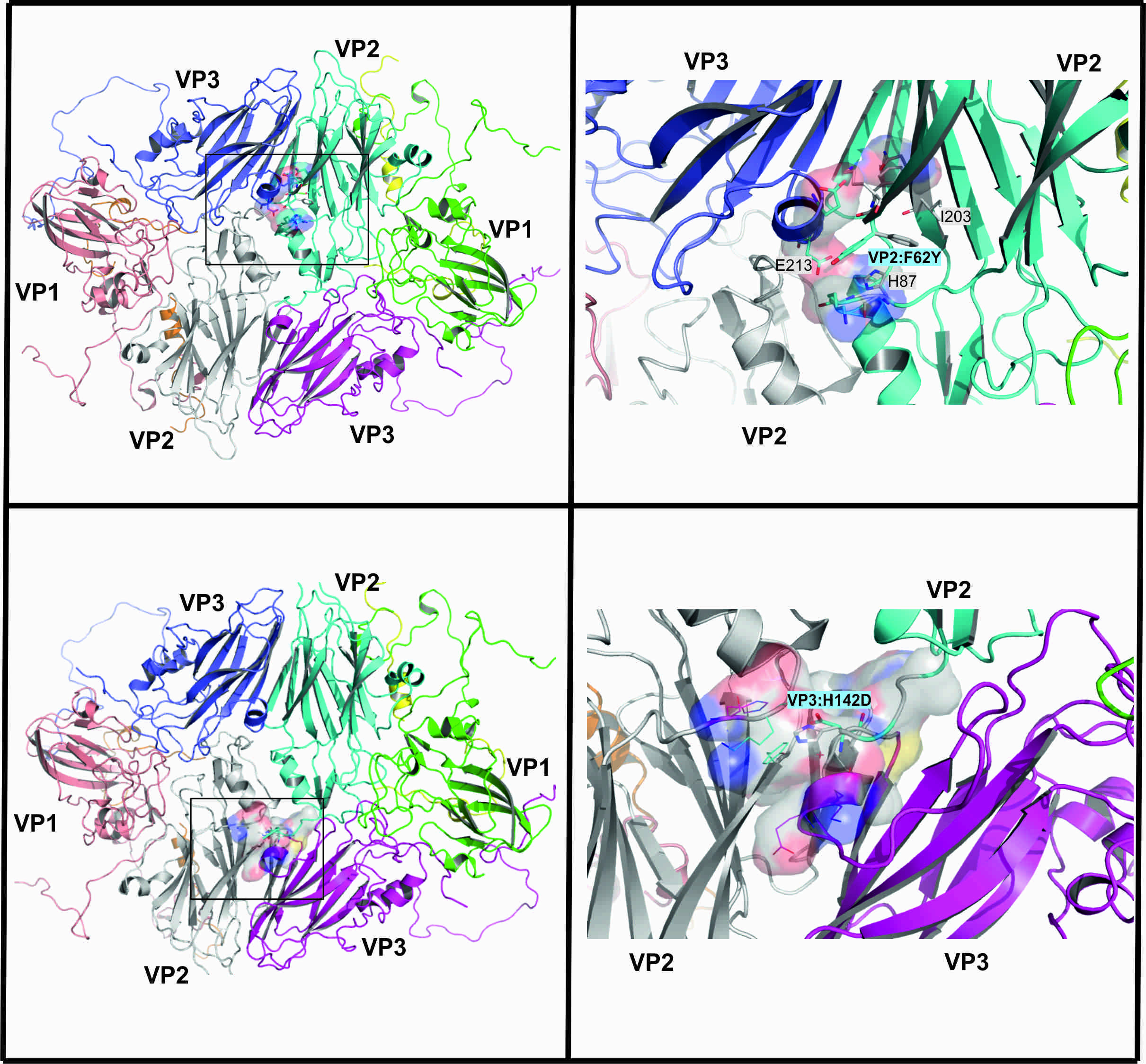
**

**Supplementary Figure 1: Demonstration of stable hydrophobic interactions in the double mutant, AM-3 of FMDV serotype A using homology modelling**

The F at 62 of VP2 region of the wild type virus was substituted with Y, which was further modified by replacing H at 142 of VP3 region with D to improve the stability through hydrophobic interaction. Substitution of F with Y in the VP2 region increased the hydrophobic interactions to five. Out of five, two hydrophobic interactions involved E at 138 and D at 142 of VP3 in the neighboring pentamers (Top row). Substitution of H with D was done to reduce the electrostatic repulsion in the VP3 region. This mutation also had an advantage of increasing the hydrophobicity by attracting Y residue at 62 and K residue at 88 of VP2 of neighboring pentamer (bottom row). Collectively, the predictions revealed that AM-3 can hold the pentamer in the native form to prevent the capsid dissociation at high temperature. Images in the second column are enlarged images of the first column.

**
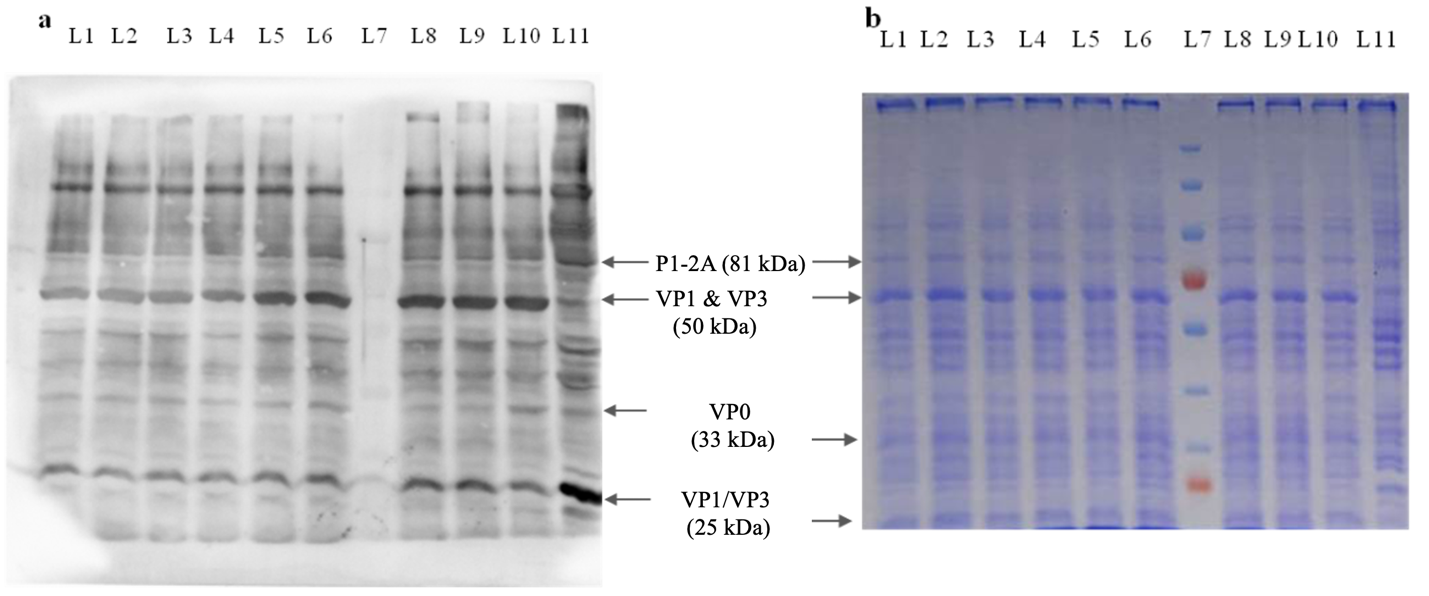
**

**Supplementary Figure 2.1: Demonstration of virus-like-particles (VLPs) of FMDV serotype A expressed in baculovirus by immunoblot**

In the un-cropped image Lanes 1 through 6 indicates mutants, AM-1 to AM-6, respectively. Lane 7 indicates the molecular marker. Lanes 8 to 10 represent AM-7, AM-8 and wild type of FMDV serotype A, respectively. Lane 11 indicates the A-WT VLP. A band at 25 kDa corresponds to VP1/VP3, while 33 kDa band corresponds to VP0 and 81 kDa band corresponds to P1-2A indicating the expression of VLPs. Panel a and b represent the Immunoblot and SDS PAGE images, respectively.


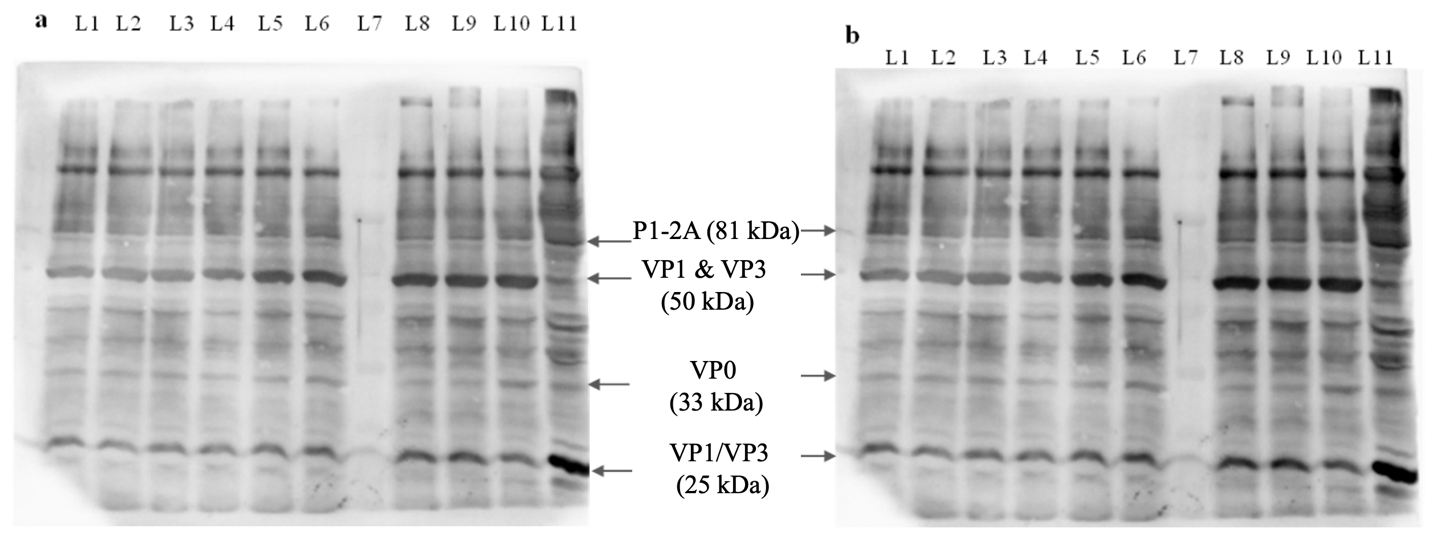


**Supplementary Figure 2.2: Demonstration of virus-like-particles (VLPs) FMDV serotype A expressed in baculovirus by immunoblot at different exposure**

Panel a and b indicate immunoblot taken at two different exposure time such as, 2.5 min and 2 min, respectively.


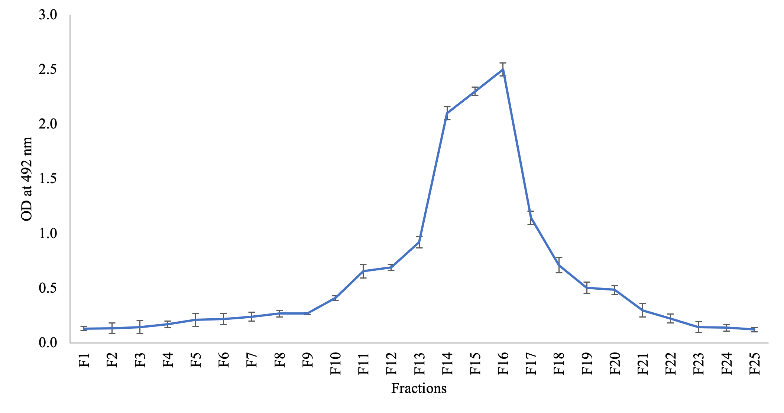


**Supplementary Figure 3: Analysis of ultracentrifuge fractions in S-ELISA**

Lysates of Tn5 cells infected with the AM-3 mutant were loaded onto a 15 - 45 % sucrose gradient. Fractions were collected and analyzed by S-ELISA. Fractions containing empty capsids (fraction 14 - 16 showed higher O.D in S-ELISA) compared to other fractions. The experiments were conducted four times (n=4) and mean with error bar were given in the histogram.


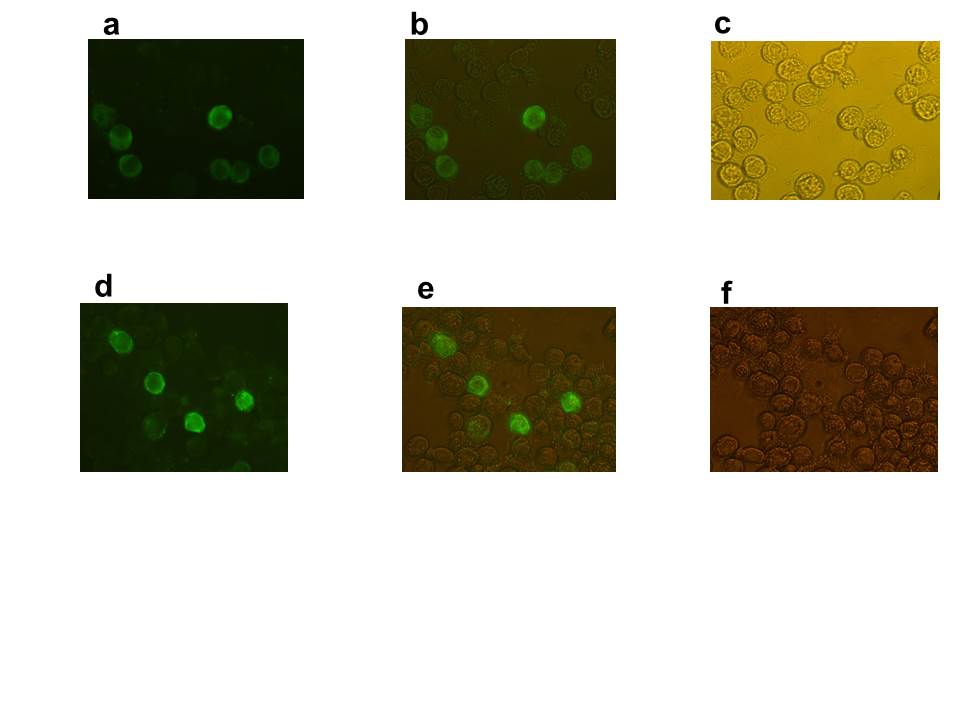


**Supplementary Figure 4: Antigenic profiling of stable AM-3 and wild type virus like particles (VLPs) by immunofluorescence assay**

The first row (a, b and c) indicates the fluorescence images of A-WT VLPs, while the second row (d, e and f) indicates the fluorescence images of AM-3 VLPs. The first, second, and third columns indicate fluorescence images, superimposed images (fluorescence and phase-contrast), and phase-contrast images, respectively. Indirect immunofluorescence assay was performed with FMDV 146S specific neutralizing monoclonal antibody and Alexa flour 488 tagged anti-mouse antibodies was used as the secondary antibody. Green fluorescence in d indicates that the mutation did not affect the antigenic site in AM-3.

**
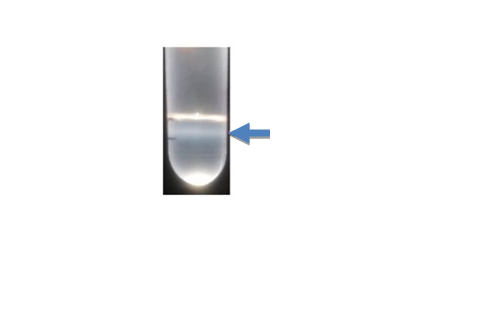
**

**Supplementary Figure 5: Demonstrating the purity of 146S antigen by cesium chloride ultracentrifugation**

Briefly, 4 mL each of two different gradients of cesium chloride such as 1.42 and 1.38 g/mL was added to 18 mL polycarbonate ultracentrifuge tube on which 10 mL of inactivated FMDV 146S antigen was layered. The tube was run at 1,35,091 g for 16 h to purify 146S which appeared as a clear white band (blue arrow). Ultraviolet spectrophotometry was used to assess purity at 259/239. The concentration was calculated by absorbance at 259 nm multiplied by the extinction coefficient, 131. The concentration of 146S was 188 µg/mL. The 146S was subjected to a two-fold dilution from 8 µg to 4 ng to construct the standard curve to quantify the VLPs concentration by S-ELISA.


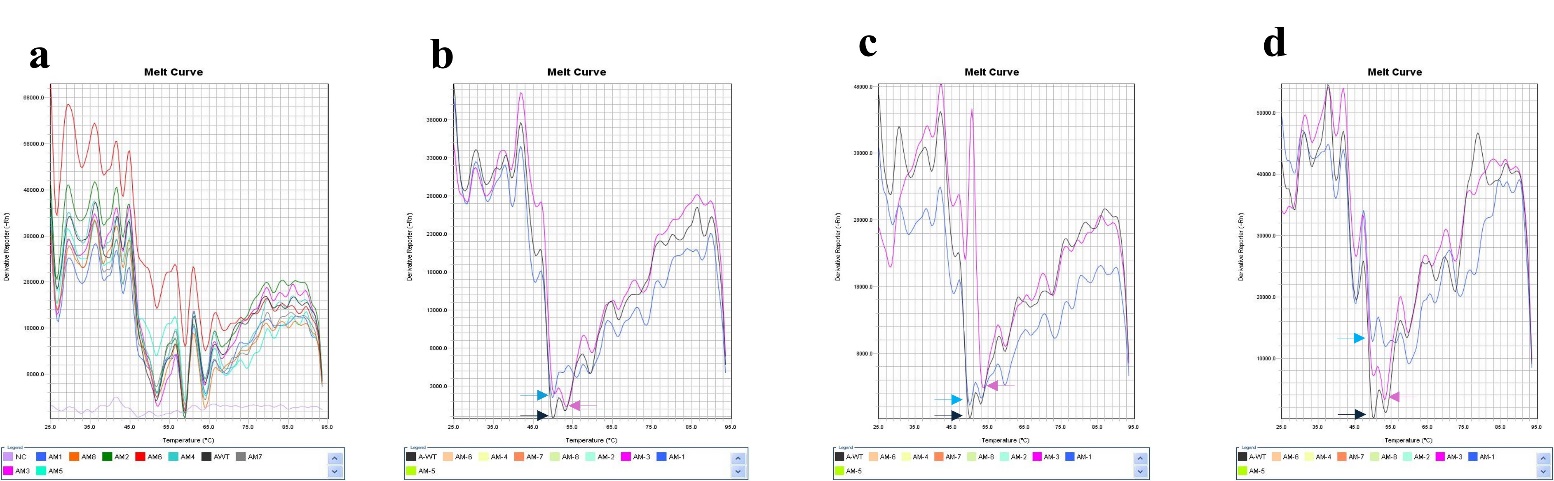


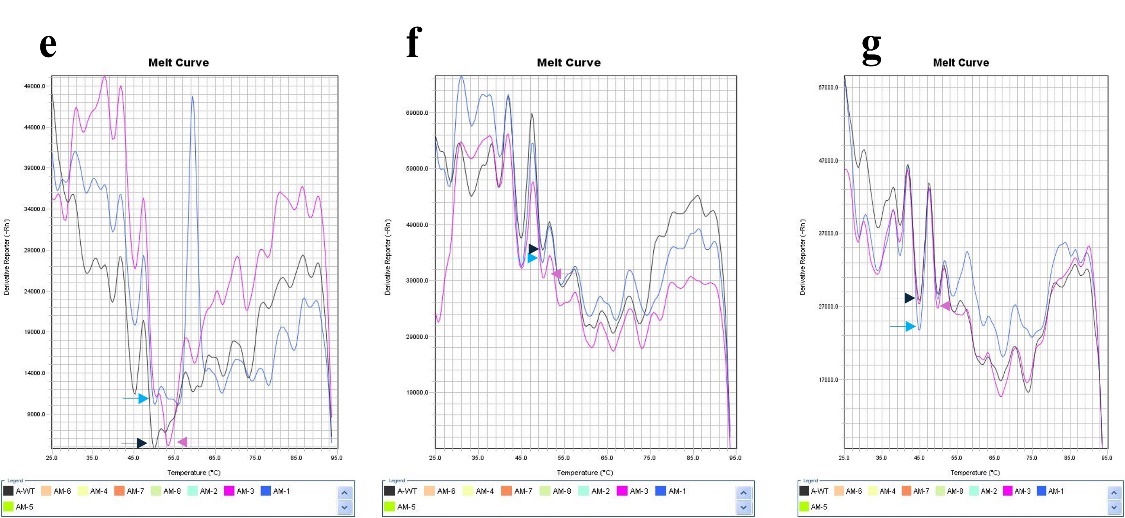


**Supplementary Figure 6: DSF assay showing the T_m_ curve of the VLPs of the FMDV serotype A**

Thermostability of wild type and mutant VLPs were analyzed by DSF assay. The Tn5 cells expressed VLP were pelleted over 20% sucrose by ultracentrifugation at 1, 31,101 g for 5 h at 10°C. The concentrated VLPs were quantified by S-ELISA with known FMDV 146S standard antigen and 4 μg of VLPs each were used for the DSF assay. SYPRO orange fluorophore served as a signal at a concentration of 5X. Melt curve analysis showing the Tm of the VLPs before exposure to high temperature (**a**), at 37 ºC for 30 min (**b**), at 37 ºC for 60 min (**c**) at 45ºC for 30 min (**d**), at 45 ºC for 60 min (**e**), at 56ºC for 30 min (**f**) and at 56ºC for 30 min (**g**). Pink, blue and black arrows indicate the Tm of AM-3, AM-1 and A-WT, respectively (n=3/mutant/time point).

**
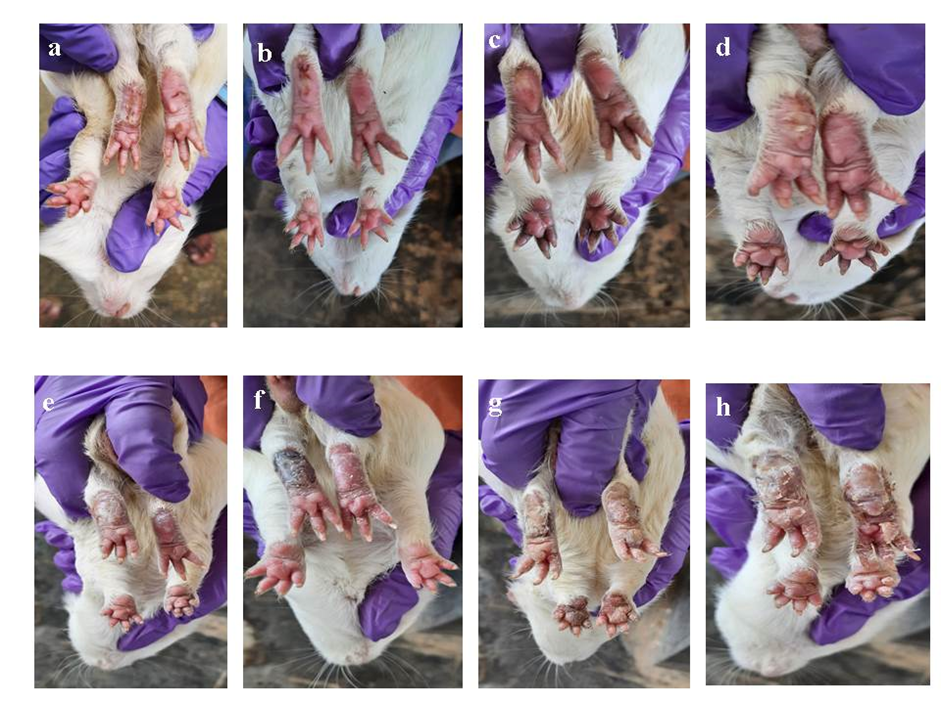
**

**Supplementary Figure 7: Demonstration of protected and non-protected guinea pigs after challenge based on lesion scoring of footpads**

A male Dunkin Hartley guinea pig weighing around 500 to 530 g was used for dose response and immunogenicity study of AM-3 VLPs. Animals were bled on day 0 vaccination to confirm the seronegativity. The protection level of dose and immunogenicity was determined by challenging the animal with serotype A 100GPID_50_ virus on day 56 dpv. The final lesion scoring was taken on day 10 post-challenge.

Figures **a, b, c and d** indicate no lesion in all four limbs. **e, f, g and h** indicate mild swelling on the right hind limb, mild swelling and vesicle formation on all four limbs, moderate vesicle formation and severe rupture of skin, respectively. a through d were considered as protected whereas; e through h were considered as non-protected.

**Supplementary Figure 8: Percentage inhibitory values in FMDV 3AB blocking ELISA with protection status among different antigen groups**

The relation between the protection status among the four groups and % inhibition value of 3AB indirect ELISA was modeled by Kaplan-Meier survival curve where non-protection was considered as event (1) while protection was considered non-event (0). The mantel-Cox log rank test indicated a significant difference in the protection among the four groups (P<0.0001). The protection % of thermostable AM3 group was comparable with that of the positive control group which was administered 146S (P>0.05).
